# Excitotoxic Insult Results in a Long-Lasting Activation of CaMKIIα and Mitochondrial Damage in Living Hippocampal Neurons

**DOI:** 10.1371/journal.pone.0120881

**Published:** 2015-03-20

**Authors:** Nikolai Otmakhov, Elena V. Gorbacheva, Shaurav Regmi, Ryohei Yasuda, Andy Hudmon, John Lisman

**Affiliations:** 1 Biology Department, Brandeis University, Waltham, Massachusetts, 02454, United States of America; 2 Max Planck Florida Institute, One Max Planck Way, Jupiter, Florida, 33458, United States of America; 3 STARK Neuroscience Research Institute, Indiana University School of Medicine, 950 West Walnut Street, Research Building II, Room 480, Indianapolis, Indiana, 46202, United States of America; Albany Medical College, UNITED STATES

## Abstract

Over-activation of excitatory NMDA receptors and the resulting Ca^2+^ overload is the main cause of neuronal toxicity during stroke. CaMKII becomes misregulated during such events. Biochemical studies show either a dramatic loss of CaMKII activity or its persistent autonomous activation after stroke, with both of these processes being implicated in cell toxicity. To complement the biochemical data, we monitored CaMKII activation in living hippocampal neurons in slice cultures using high spatial/temporal resolution two-photon imaging of the CaMKIIα FRET sensor, Camui. CaMKII activation state was estimated by measuring Camui fluorescence lifetime. Short NMDA insult resulted in Camui activation followed by a redistribution of its protein localization: an increase in spines, a decrease in dendritic shafts, and concentration into numerous clusters in the cell soma. Camui activation was either persistent (> 1–3 hours) or transient (~20 min) and, in general, correlated with its protein redistribution. After longer NMDA insult, however, Camui redistribution persisted longer than its activation, suggesting distinct regulation/phases of these processes. Mutational and pharmacological analysis suggested that persistent Camui activation was due to prolonged Ca^2+^ elevation, with little impact of autonomous states produced by T286 autophosphorylation and/or by C280/M281 oxidation. Cell injury was monitored using expressible mitochondrial marker mito-dsRed. Shortly after Camui activation and clustering, NMDA treatment resulted in mitochondrial swelling, with persistence of the swelling temporarily linked to the persistence of Camui activation. The results suggest that in living neurons excitotoxic insult produces long-lasting Ca^2+^-dependent active state of CaMKII temporarily linked to cell injury. CaMKII function, however, is to be restricted due to strong clustering. The study provides the first characterization of CaMKII activation dynamics in living neurons during excitotoxic insults.

## Introduction

Most animal studies of stroke require that treatment be applied before or shortly after the stroke event. This could be a reason why many clinical trials for stroke treatment based on these studies have been negative [1]. Recently, it was shown that inhibitors of activated CaMKII significantly decrease neuronal damage even if applied one hour after an experimental stroke *in vivo* or 2–6 hours after an excitotoxic insult in neuronal cultures [2,3,4]. The results implicate that an active state of CaMKII may last for many hours after stroke and play a significant role in delayed brain toxicity. Understanding the mechanisms of this role is crucial for developing a new approach in “after-stroke” treatment.

Over-activation of the excitatory NMDA receptors and the resulting Ca^2+^ overload is the main cause of neuronal toxicity during stroke [5]. CaMKII is one of the major Ca^2+^ sensors in neurons and can operate as a molecular switch [6]. Under basal conditions, the regulatory region of the kinase binds to and inhibits the catalytic region. When intracellular Ca^2+^ rises, Ca^2+^/CaM binds to the regulatory region, leading to a separation of the regulatory and catalytic regions rendering kinase activation. Autophosphorylation of the regulatory region at T286 prevents regulatory and catalytic domains from rebinding and leads to persistent kinase activation even after Ca^2+^ returns to the basal level. This Ca^2+^-independent (or autonomous) conformational state remains until T286 is dephosphorylated. Therefore, CaMKII can operate as a switch, sustaining its active conformation after a transient activation. This memory-like property has been shown to be critical for behavioral and synaptic memory [7,8,9] but has been also implicated as both a cell damaging and pro-survival force in ischemic/ excitotoxic insult [4].

Despite considerable efforts, even the time course of CaMKII activation after ischemic/excitotoxic insult remains controversial. Currently, there are two major groups of results, which are not easily reconcilable. During the first minute of ischemia, CaMKII undergoes brief increase in Ca^2+^-dependent and Ca^2+^-independent activation [10] and autophosphorylation at T286 [11,12]. Most studies of the first group described this initial phase of activation as very short (1 min) [10,13]. In the following period, a loss of Ca^2+^-dependent [10,14,15,16,17,18,19], Ca^2+^-independent [10,17,19] activity and T286 phosphorylation [19,20] was observed. Some reduced level of persistent activity and T286 phosphorylation still remained during a prolonged ischemia [10,17,21,22,23] and during the following reperfusion [19,20,22]. In contrast, the second group of studies reported a fast (3 min) and prolonged (at least 30 min) increase in CaMKII phosphorylation of T286 in the total homogenate and membrane fractions (including PSD/synaptosomal) during ischemia [11], which stayed elevated for at least 30–60 min or longer during the reperfusion [22,23,24]. Therefore, some evidence support persistent activation while others support persistent inactivation of CaMKII after an excitotoxic event.

All studies, however, agreed that, very shortly after the start of ischemia, CaMKII translocates from the cytosolic to the particulate fraction [10,16, 17,19,20,21,22,25] and, specifically, to the postsynaptic density (PSD) fraction. Imaging studies showed, that in addition, to the PSD, CaMKII gets sequestered into dense aggregates or clusters in cell somas [26,27,28,29,30]. It was suggested that the particulate fraction accumulation and clustering may serve as a protection mechanism to prevent cell from hyperphosphorylation by CaMKII [16,26]. However, long-lasting inhibition of CaMKII due to aggregation could also be deleterious to cell function and survival [3,14,31].

Based on these findings, two alternative hypotheses were put forward. One group proposed that the loss of CaMKII activity was a critical factor in the ischemic cell damage; the other group suggested that the over-activation of the kinase due to a persistent autonomous state induced cell damage. Recent results added significant support to the second hypothesis: strong evidence was presented that a persistence of CaMKII in autonomous (“active”) state was required to produce cell damage after an ischemic or excitotoxic insults. Specifically, it was shown that inhibitors of active state (CN21 or AIP), but not an inhibitor of Ca^2+^/CaM-dependent activation (KN93) of CaMKII significantly decreased cell death even if they were applied hours after an ischemic or excitotoxic insult [2,3]. One possibility, which could reconcile the apparent contradiction in results, is that the autonomous CaMKII constitutes only a fraction of the total kinase in tissue, perhaps localized at a small cell population or in subcellular domains such as synapses or intracellular organelles. These domains may contribute very little to biochemical measurements of total CaMKII activity, yet still play a significant role in cell damage after ischemia.

Therefore, to complement the biochemical data, we directly measured the changes in the activation state of CaMKII in living hippocampal neurons in slice cultures using the CaMKII activity sensor, green Camui (Camui) [32]. One advantage of this approach is that CaMKII activation state can be differentially monitored in soma, dendrites, or dendritic spines at high spatial and temporal resolution. The Camui sensor is composed of CaMKIIα tagged with dVenus and GFP fluorophores on its N and C termini, respectively. Upon CaMKII activation, the distance between the termini increases, leading to a decrease in the Forster resonance energy transfer (FRET) between the fluorophores (see also [33,34,35,36]) and to an increase in GFP lifetime [32]. Therefore, change in the fluorescent lifetime of the Camui GFP reveals transition of CaMKII between its closed (inactive) and open (active) states [32]. Previously, experiments *in vitro* and in living cultured cells clearly demonstrated an increase in Camui fluorescence lifetime upon CaMKII activation by Ca^2+^/CaM or upon its transition into an autonomous (Ca^2+^-independent) active state [32,33,34]. Following Lee at al., 2009, we will use the terms “activation” and “increase in fluorescence lifetime” interchangeably in this text. Strictly speaking, however, it is possible that, while in open “autonomous” or “active” conformational state, the enzymatic activity of Camui is inhibited due to masking of catalytic sites by an inhibitor [34] or other structural rearrangements, including CaMKII aggregation induced by self-association [28,29,37].

To estimate the downstream consequences of an NMDA excitotoxic insult on neuronal health, we monitored mitochondrial damage as a readout of cell injury associated with cell death after ischemic/excitotoxic insults [38,39,40]. Our data indicate that in living cells an excitotoxic insult results in a long-lasting Ca^2+^-dependent activation of CaMKII, with a limited impact of the autonomous kinase state. The duration of CaMKII activation was temporarily linked to the duration of mitochondrial damage. Extensive CaMKII clustering, however, suggests a dramatic loss of normal kinase function.

## Materials and Methods

### Slice culture preparation and DNA transfection

Hippocampal slice cultures were prepared from Long-Evans rats at P6–9 and were maintained for 11–14 days as described earlier [41] before cDNA transfection. Transfection of cDNA into CA1 pyramidal cells was performed by single-cell electroporation [8,42,43]. Two slightly different variants of the wild-type (WT) Camui and its mutants were used. The differences were due to different methods of the fluorophore subcloning. Specifically, variant 1 Camui had an additional 26 amino acid sequence (SGLRSRAQASNSAVDGTAGPGSTGSR) at the end of GFP. Although both versions produced qualitatively similar results, the quantitative results for the duration of persistent activation were slightly different, and the data for each type of Camui are therefore presented separately. K42R and I205K mutants were prepared using variant 1 Camui and T286A/CM280/281VV, or CM280/281VV mutants were prepared using variant 2 Camui. For control experiments, Camui of the same variant as corresponding mutants were used. Control and mutant experiments were performed on the same or on a consecutive day using slices from the same preparation. Camui reactivity and redistribution in response to glutamate uncaging in spines were extensively characterized previously [32]. In that study, both Camui variants produced similar results, and all data were pooled together. In some experiments together with Camui, we also coexpressed mito-dsRed cDNA (Clontech Lab., Inc.) to label mitochondria. The study was carried out in strict accordance with the recommendations in the Guide for the Care and Use of Laboratory Animals of the National Institutes of Health. The protocol was approved by the Committee on the Ethics of Animal Experiments of the Brandeis University (PHS Animal Assurance #: A3445-01; Protocol #13001)

### Imaging and image analysis

One or two days after electroporation, slices expressing Camui were cut out of the 6-well insert membranes used for their support and were transferred to the glass bottom of a custom-made recording/imaging chamber placed on a custom-made motorized stage. Slices were completely submerged in a circulating ACSF (flow rate 2.5–3 ml/min) of the following composition (in mM): 124 NaCl, 2.5 KCl, 4 CaCl_2_, 4 MgCl_2_, 1.25 NaH_2_PO_4_, 26 NaHCO_3_, 20 Dextrose, 0.001 TTX, balanced with 95% O_2_ and 5% CO_2_, pH 7.4 at room temperature (22C°–24C°). Slices were equilibrated in these conditions for at least 30 min before imaging. Imaging was performed using a custom-made two-photon microscope system, as described in [32,44]. The system was equipped with a Coherent XR two-photon laser, two fast PMTs (H7422-40), SPC150 FLIM board (Becker- Hickl, DE), Pockels cell (Conoptics, CT), and 60× 1.0 NA WI (Olympus, JA) objective. Time-lapse FLIM imaging and analysis were performed using custom software, as described in [32,44]. GFP and mCherry were excited by mode-locked IR at 920 nm. Green and red emissions were separated by DCXR 565 nm dichroic and HQ 510/70 nm and ET630/60—P blocking filters (both from Chroma technology). Normally, a Z stack of 5–7 focal steps (1 μm) was acquired to image a dendritic segment. Each focal plane image (128 × 128 pixels at 0.1 μm pixel size) was averaged 30–60 times. Weak laser intensity (~1.0 mW under the objective) was used to minimize bleaching and photodamage. Time-lapse imaging was done every 2–8 min. Green PMT signal was recorded in single-photon counting mode (SPC), and two types of images were constructed: one—presenting averaged number of photons per pixel (SPC image) and the second—presenting averaged single photon lifetime per pixel (FLIM). Red fluorescence was recorded in standard integral mode. Analysis was performed from the maximal projection images of each stack. Background was subtracted from SPC image to determine cell edges, and then ROI were drawn around individual spines and along the dendrite or at a proximal part of cell body that did not include the nucleus. Special care was taken to avoid bright spots on the dendritic shaft of the SPC image, which could represent spines or occasional protein aggregates. Further analysis was done using integral SPC of spine ROIs and average SPC of dendritic or somatic ROIs. Only ROI pixels positioned within the cell border were taken into account in these calculations. Average fluorescence lifetime in spine, dendritic, and somatic ROIs were calculated using FLIM projection images. Morphological changes were analyzed qualitatively by visual criteria.

Because fluorescence intensity (SPC) of Camui donor fluorophore (GFP) slightly quenches due to FRET, the following correction was used for each ROI: Corrected Intensity = Measured Intensity * (tau_GFP/tau_m), where tau_GFP = 2.6 ns and tau_m = measured average Camui tau.

### Excitotoxic insults

Previous data showed that treatment of hippocampal neurons with 10μM of NMDA in normal Mg^2+^ for 24 hours or with larger concentrations (300 μM) for 5 min produced delayed cell death in about 50% −80% of neurons [2,45,46]. Because cells depolarization during ischemia releases Mg^2+^ block from NMDA receptors, we applied NMDA (25 μM for 5–8 min, as mentioned in the text) in ACSF containing 0.3 mM of Mg^2+^. In a few initial experiments on dendritic spines, lower concentrations of NMDA (10 and 15 μM) or lower Mg^2+^ (0 mM) in the ACSF were used, but because the effects at different conditions were similar, all results were pooled together. For summary figures and statistical analysis, only experiments with 0.3 mM Mg^2+^ in ACSF were included. Washout was performed with standard ACSF supplemented with NMDA receptor antagonist D, L APV (100 μM). The first 30 min of the washout was performed using ACSF flow without recirculation to ensure complete removal of drugs from the slice imaging chamber. ACSF flow was then recirculated for an additional 30–40 min. TTX (1μM) was routinely used during the whole lengths of experiments to prevent cell spiking.

### Drugs

Stock solutions of NMDA (Sigma, 20 mM), D, L-APV (Sigma, 20 mM) and TTX (Abcom Biochemicals, 2 mM) were prepared in deionized water, and their aliquots were kept frozen until use.

### Statistical analysis

All data were presented as mean ± SEM. Student’s t-test (Excel, 2010) or “One-Way Analysis of Covariance" (http://vassarstats.net/vsancova.html) ANCOVA (Excel) tests were used to calculate statistical significance between sets of data. Noise level of fluorescence lifetime recordings was calculated as 95% confidence interval from data obtained in ~180 minute time-lapse imaging of cell somas (baseline control).

## Results

### Camui activation state and expression pattern at basal conditions

To study how excitotoxic insults affect CaMKII activity in living cells, we transfected pyramidal CA1 neurons in hippocampal slice cultures with cDNA of CaMKII activity probe, Camui. Camui green fluorescence was recorded in both single photon counting (SPC) and lifetime modes; and the respective two types of images were constructed online. SPC images represent spatial distribution of Camui fluorescence intensity (average number of single photons per pixel) in cell. This measure is proportional to the protein amount of Camui within a subcellular region. FLIM images depict average fluorescence lifetime of green photons of Camui (time between the fluorophore excitation and emission) expressed in nanoseconds per pixel. The fluorescence lifetime reflects different conformational states of CaMKII: longer lifetime corresponds to more open (“active”) conformation (see “Introduction” for additional explanation).

**Fig 1:**
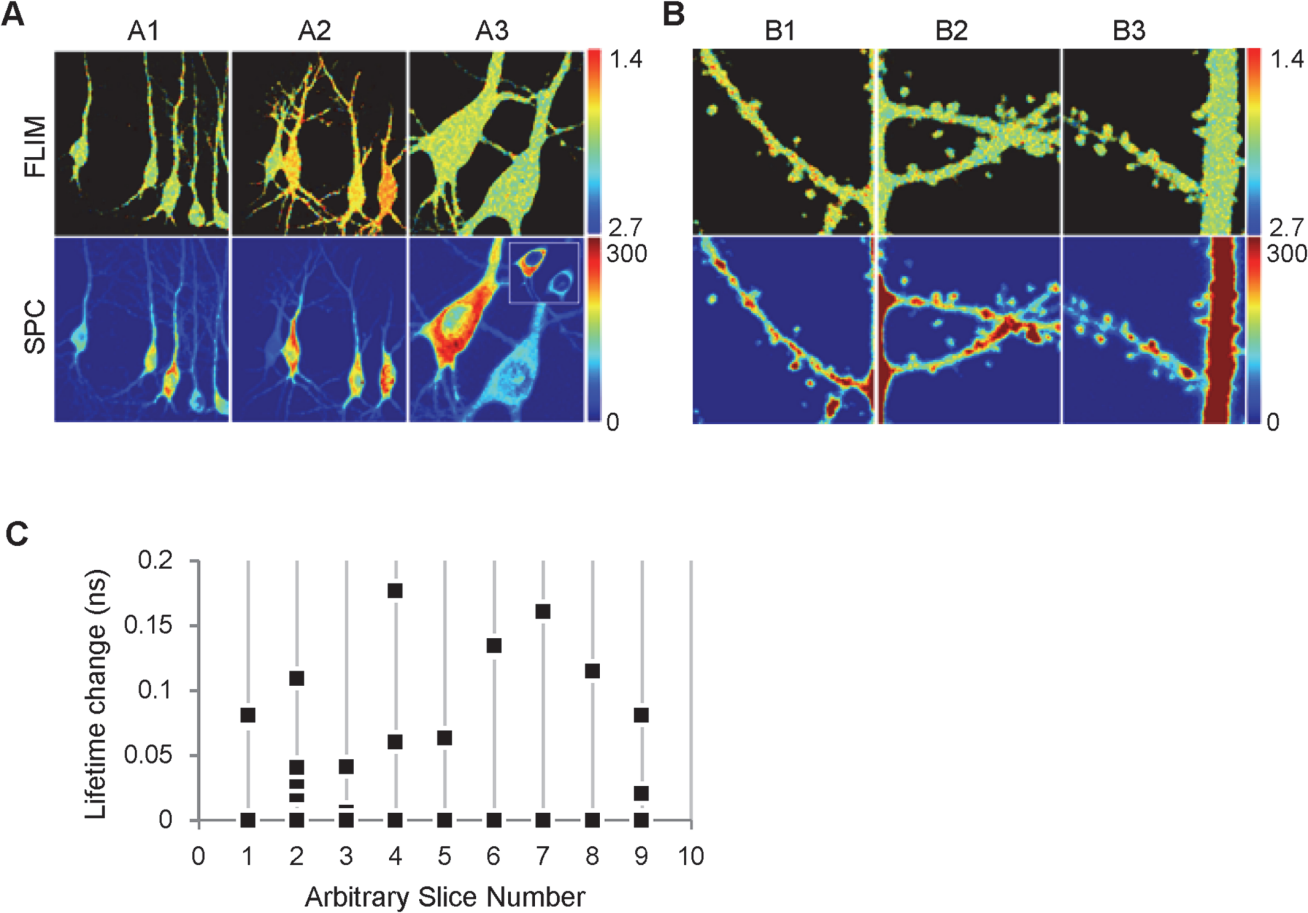
Basal Camui activity is uniform across spines, dendritic shafts, and somas of the same cell, but differs between cells. (A) Representative low-magnification (A1-3) and high-magnification (B1-3) images of Camui fluorescence lifetime (FLIM) and single-photon counting (SPC) obtained in different experiments. Brighter colors correspond to lower level of Camui activation (shorter lifetime) in FLIM images and to higher Camui green fluorescence (more photons) in SPC images. Camui activity could be similar (A1) or very different (A2-3) in different cells. The insert (A3, SPC) shows a single focal plane image through the middle of cell bodies, demonstrating no Camui expression in the cell nuclei. Note that (B1-3) images are not zoomed in regions of images shown in (A1-3). Image sizes: (A1-A2): 60 μm, (A3): 20 μm, (B1-3): 12 μm; Pseudo-color scales, ns (FLIM) and a.u. (SPC). (C) Graph demonstrates that different cells in the same slice may have very different fluorescence lifetime in somatic region, suggesting different level of Camui activation (n = 26). Each data point represents a value of fluorescence lifetime for each cell in the same image (slice) normalized to the minimum fluorescence lifetime value in that image.

Camui spatial distribution in cell bodies and dendrites was, in general, homogeneous except that almost no protein was visible in the cell nuclei (Fig. 1A, SPC), as expected [32,43]. Comparison of the fluorescent lifetime of Camui in neighboring cells revealed a large variability (0.088 ± 0.01 ns, 9 slices, 26 cells, Fig. 1A2, C). Previous studies reported that the CaMKII amount in spines correlated with spine size and synaptic strength [47]. Interestingly, in the current study, we find that although Camui amount was also quite variable between spines [41,47], the activation state of Camui in different spines was similar (R^2^ = 0.06; Fig. 1B, S1 Fig. panel B and S1 Text). Furthermore, the basal level of CaMKII activation in spines and in adjacent dendritic shaft of the same dendritic branch was also similar (1.99 ± 0.01 ns in spines and 1.97 ± 0.02 ns in dendritic shafts, 27 branches, p > 0.05). Likewise, we observed no difference between basal Camui activation in cell somas and dendrites (1.99 ± 0.03 ns in somas, n = 26 and 1.97 ± 0.02 ns in dendritic shafts, n = 27 respectively, p > 0.05).

Thus, although basal Camui activation significantly varied even between neighboring cells, it was similar in all characterized cellular compartments (spines, dendritic shafts, and cell somas) of individual cells.

### In neuronal dendrites, NMDA challenge produces either persistent or transient Camui activation with concomitant redistribution to spines

After 30 min of baseline imaging, slices were subjected to bath-applied NMDA (25 μM) for 5 min while time-lapse imaging continued every 2–8 min for at least 40–60 min. The Mg^2+^ concentration of the ACSF during the NMDA application was reduced to 0.3 mM to partially release Mg^2+^ block of NMDA receptors, which, during an actual ischemic episode, occurs as a result of cell depolarization. The washout of the NMDA was performed using ACSF with standard Mg^2+^ and 100 μM of APV (an NMDA receptor antagonist). APV was included to facilitate termination of NMDA effect because the cellular glutamate uptake mechanism cannot remove this glutamate analog.

**Fig 2:**
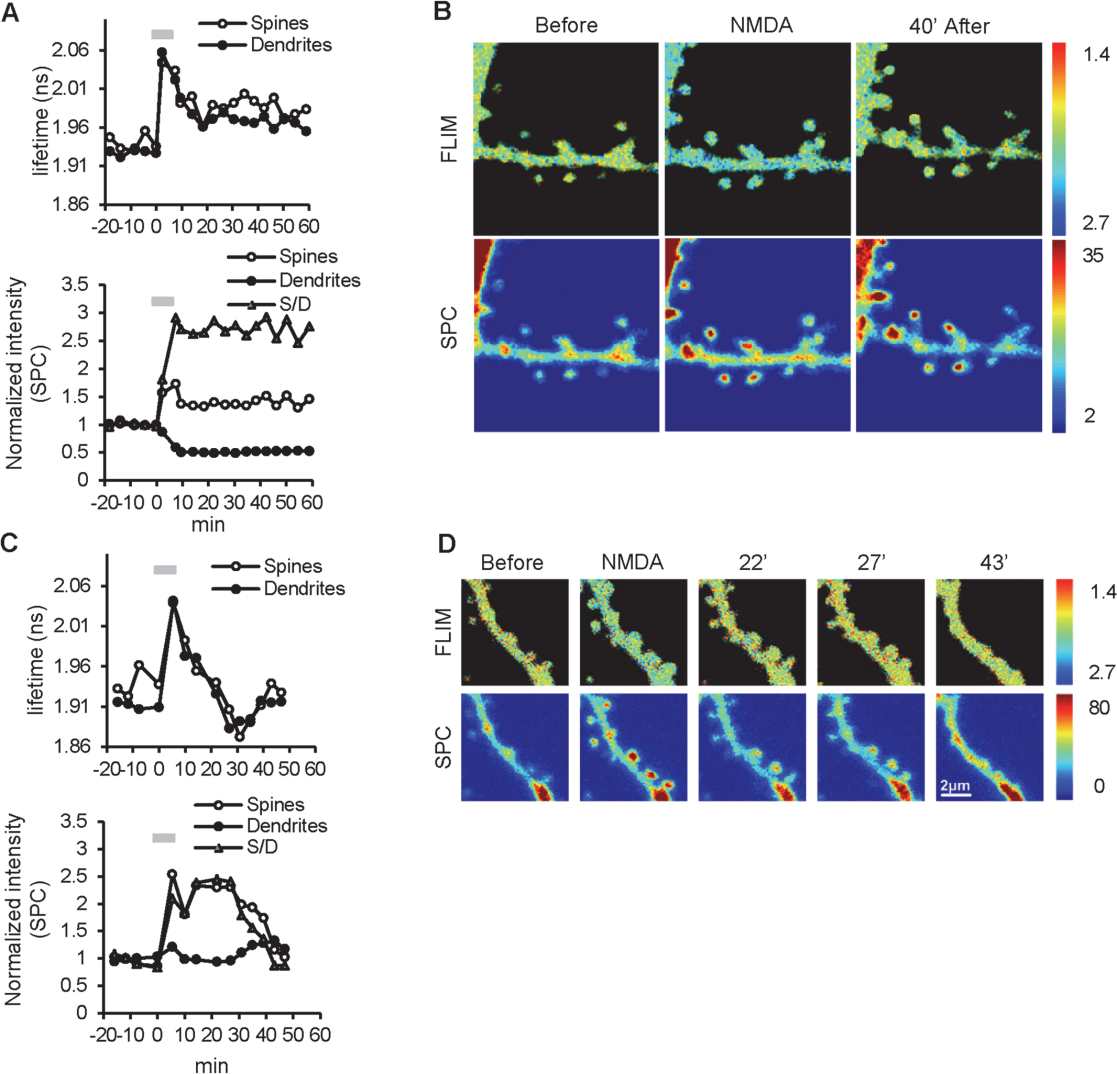
Examples of Camui reactivity in dendrites in response to 5 min NMDA treatment. (A) *Top panel*: NMDA application (grey bar) resulted in a persistent activation of Camui (increased fluorescent lifetime) in both spines (open circles) and dendritic shafts (filled circles), which remained above the baseline for at least 55 min after the washout; *bottom panel*: Corresponding fluorescence intensity persistently increased in spines (open) and decreased in dendritic shafts (filled), resulting in a dramatic increase in the spine/dendrite fluorescence ratio (triangles). (B) Images of FLIM (top) and fluorescence intensity (SPC, bottom) before, during NMDA application, and 40 min of washout for the experiment shown in (A). (C) *Top panel*: NMDA application (grey bar) resulted in a transient increase in fluorescence lifetime of Camui in both spines (open circles) and dendritic shafts (filled circles), which decayed to below baseline level by 30 min of washout; *bottom panel*: Corresponding fluorescence intensity in spines (open circles) transiently increased while remaining stable in dendritic shafts (filled circles), resulting in a transient increase in the spine/dendrite fluorescence ratio (triangles). (D) FLIM and SPC images before, during NMDA application, and at 22, 27, and 43 min of washout for the experiment shown in (C). Note that many spines became smaller or completely eliminated at the end of the experiment (43 min of washout). Pseudo-color scales, ns (FLIM) and a.u. (SPC).

In this series of experiments, one dendritic segment per slice was studied in an experiment. Transient NMDA application produced two major types of Camui responses. In group I (7 of 16 experiments), Camui activity increased during the NMDA application and maintained this activated state for the entire recording (see a representative example in Fig. 2A, B). In the second major group (group II, 5/16) NMDA stimulation produced only transient Camui activation, with activity returning to baseline level or below baseline within 20–30 min (an example in Fig. 2C, D). In both groups, the amount of Camui in spines (estimated by GFP fluorescence intensity) increased during the NMDA treatment, consistent with previous reports [48]. Camui amount in group I dendrites decreased concomitantly with its increase in spines, suggesting a translocation of Camui from dendrite to spines similar to what was observed for endogenous CaMKII in ischemic conditions [49]. In group II, Camui amount in dendrites did not significantly change, suggesting that the increase in Camui fluorescence in spines was due to an increase in spine size or local translation rather than its translocation from dendritic shafts. In general, the increase in the spine fluorescence was concomitant with the change of Camui activity: persistent in group I but transient in group II (Fig. 2A, C, *bottom panels*). In group II, however, the increase of Camui fluorescence in spines often lasted longer than Camui activation (Fig. 2C and S2 Fig.).

In addition to these two major groups, two other types of Camui reactivity following NMDA treatment were observed (S3 Fig., S1 Table). Fluorescence lifetime measurements were very noisy in group III (3/16) and showed no change in group IV (1/16). Camui fluorescence intensity decreased in both spines and dendrites in group III but in group IV it only decreased in spines while dendritic fluorescence remained stable. Also significant cell swelling/shrinkage was evident in both of these groups (S3 Fig., panel C), whereas cells in groups I and II displayed little or no dendritic swelling or shrinkage (Fig. 2, S2 Fig. and S4 Fig.).

**Fig 3:**
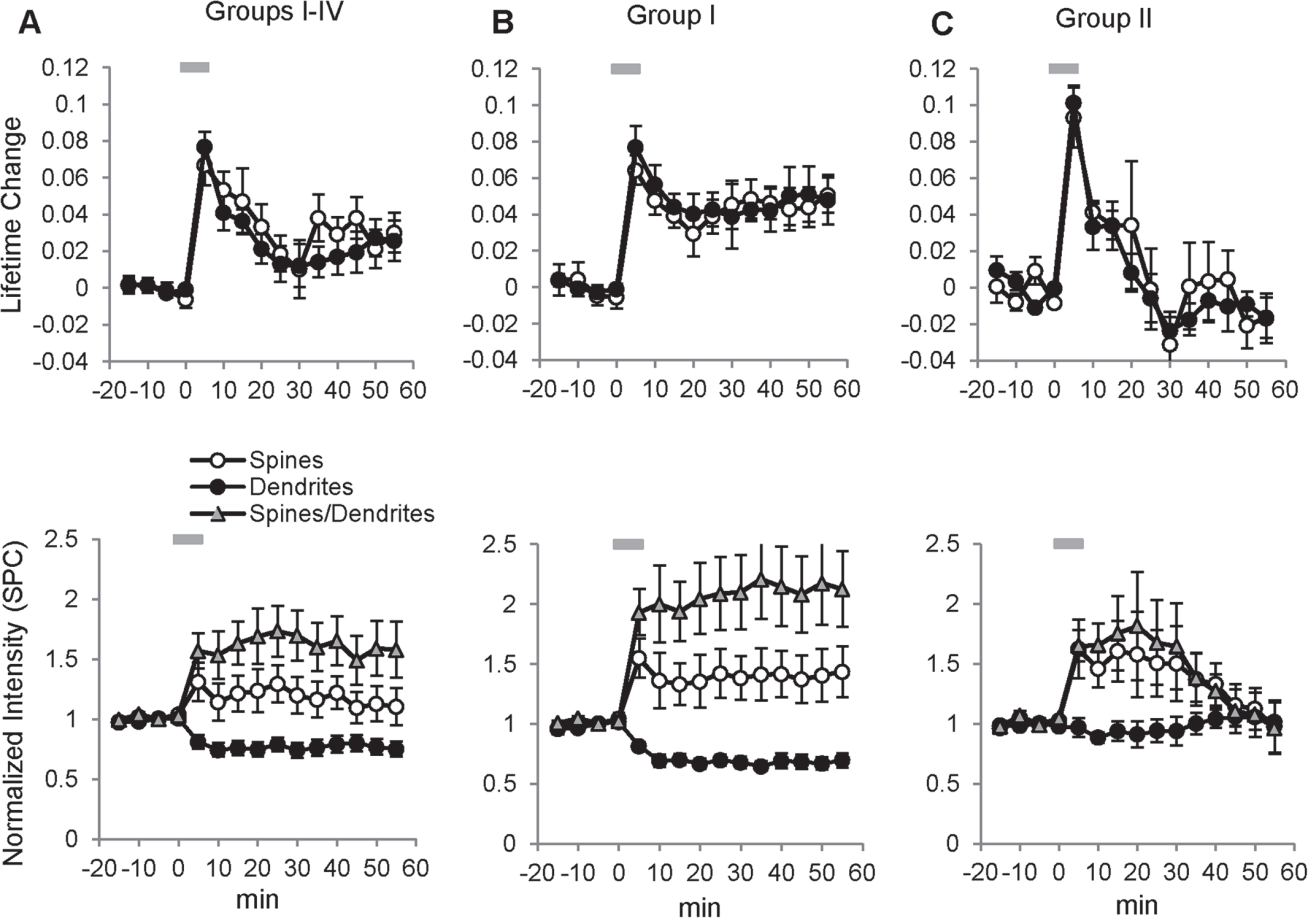
Summary of Camui reactivity in dendrites in response to 5 min NMDA treatment. (A) *Top panel*: Average of all 16 experiments (Groups I–IV); NMDA application (grey bar) resulted in a persistent increase in fluorescence lifetime of Camui in both spines and dendritic shafts, which remained elevated for at least 55 min after the washout; *Bottom panel*: fluorescence intensity of Camui in spines persistently increased (open circles), while the fluorescence in dendritic shaft concomitantly decreased below the baseline (filled circles), resulting in a dramatic increase in the spine/dendrite fluorescence ratio (triangles). (B) *Top panel*: summary plots of group I (7/16) experiments showing that application of NMDA (grey bar) resulted in a persistent increase in fluorescence lifetime of Camui in both spines and dendritic shafts (p < 0.05 for both) and remained elevated for at least 55 min (p < 0.05 for both) after the washout; *bottom panel*: fluorescence intensity of Camui in spines persistently increased (p < 0.05, open circles), while the fluorescence in dendritic shafts concomitantly decreased below the baseline, (p < 0.05, filled circled), resulting in a dramatic increase in the spine/dendrite fluorescence ratio (triangles). (C) *Top panel*: summary plot of 5/16 experiments (group II) showing that NMDA application (grey bar) produced only a transient increase in fluorescence lifetime of Camui in both spines and dendritic shafts (p < 0.05 for both) immediately after the NMDA application, but decreased in spines and in dendrites at 30 min of the washout; *bottom panel*: fluorescence intensity of Camui in spines remained elevated (open circles) for at least 30 min of NMDA washout, but then gradually retuned to the baseline by 45 min. The Camui fluorescence in dendrites (filled circles) did not change significantly, and the spine/dendrite fluorescence ratio (triangles) followed the general time course of the change in the fluorescence in spines.

The summary data for all 16 experiments (Groups I–IV) are shown in Fig. 3A. Despite different Camui reactivity patterns in different groups, overall NMDA treatment produced a small but significant persistent elevation in Camui fluorescence lifetime (0.034 ± 0.012 ns in spines and 0.025 ± 0.016 ns dendrites, p < 0.001 for both) in these combined data. The combined data also showed that the Camui fluorescence intensity persistently increased in spines (~ 20%; p < 0.001) and decreased in the dendritic shafts (~ 20%; p < 0.001), resulting in a dramatic increase (60%) in the spine/dendrite fluorescence ratio. Averaged data for groups I and II are shown in Fig. 3B,C and look very similar to the previously described representative examples from these groups (Fig. 2). Notably, for all four groups, there was no statistical difference between general dynamics of fluorescent lifetime changes in spines and in dendritic shafts (S3 Text).

Therefore, NMDA insults produced either persistent or transient Camui activation in dendrites and spines with, in general, concomitant redistribution of Camui to spines. In dendrites with transient Camui activation, however, the redistribution of Camui amount on average lasted longer than its activation, suggesting that the maintenance of Camui activation and its protein redistribution may have different regulation or phases.

### Camui activation by NMDA challenge is uniform across multiple subcellular compartments: soma, dendrites, and spines

**Fig 4:**
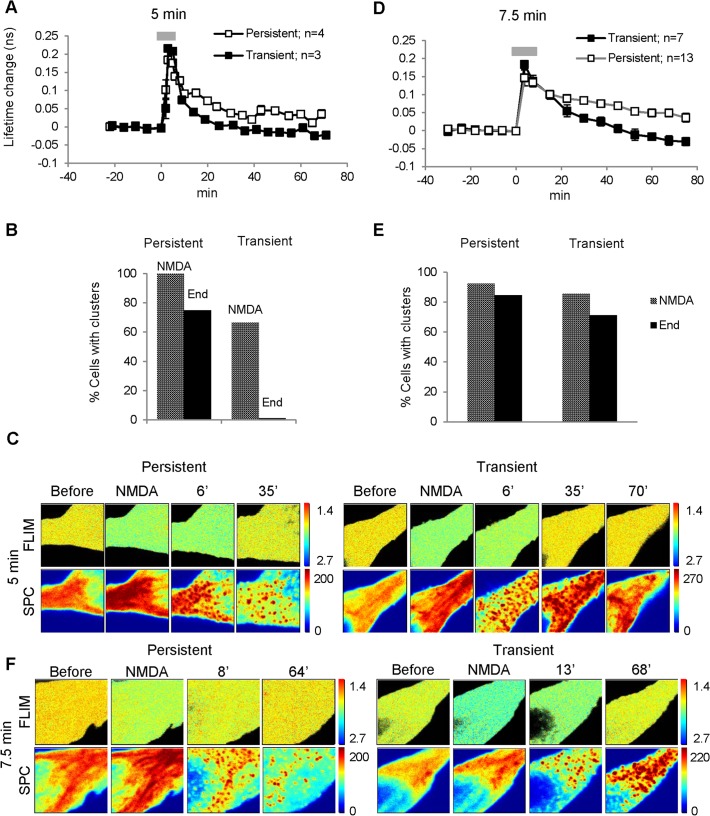
Camui activation and redistribution in neuronal somas after 5 min and 7.5 min NMDA treatment. In neuronal somas, Camui activation (A) in response to 5 min NMDA treatment was also either persistent (open squares) or transient (filled squares). (B) Camui clustering was persistent in cells with persistent Camui activation (75% of cells maintained clusters until the end of experiment) but was only transient in cells with transient activation (0% maintained clusters). (C) Images of Camui fluorescence lifetime (FLIM) and its fluorescence intensity (SPC) for groups with persistent and transient Camui activation taken before, during, and at different times (in minutes) after the 5 min NMDA treatment. Note that there was no direct correlation between the magnitude of the Camui activation and clustering: clustering remained after Camui activation returned to the baseline (Persistent, 35 min). (D) Extended (7.5 min) NMDA treatment produced sustained somatic Camui clusters, which persisted longer than Camui activation. Summary graph showing persistent (open squares, 13/20 cells) and transient (filled squares, 7/20 cells) Camui activation. (E) Camui clustering was persistent in both groups of cells (with either persistent or transient Camui activation). Notably, 71% of cells with transient Camui activation maintained clusters until the end of the experiment in comparison to 0% (B). (F) Images of Camui fluorescence lifetime (FLIM) and its fluorescence intensity (SPC) for groups with persistent and transient Camui activation taken before, during, and at different times (in minutes) after the 7.5 min NMDA treatment. Note that somatic Camui clusters persisted to the end of experiments in both these examples. For better contrast, the upper scale for the last SPC image was changed to 270 (C, 70') and 220 (F, 68'). Pseudo-color scales, ns (FLIM) and a.u. (SPC).

We next investigated Camui reactivity to NMDA treatment in neuronal soma. To address whether Camui activation in neuronal somas was similar or different to the activation seen in dendrites, simultaneous Camui measurements were collected from soma and dendrites of the same cell. Similar to what was observed in dendritic regions, Camui activation in the cell body after 5 min NMDA application was either persistent for more than 60 min (4/7 experiments) or transient (3/7 cells), returning to the baseline at ~25 min after initial activation by NMDA (Fig. 4A). Peaks of Camui activation were similar in both of these groups (lifetime change ~0.2 ns, p > 0.05). However, in cells with the transient activation, Camui slowly deactivated and then further dropped below the baseline level during the washout period (40–70 min), consistent with the presence of the basal level of Camui activity. Comparative Camui activation data for somas and dendrites are shown in S5 Fig. panels A, B. Again the results were grouped in respect to the type of the reactivity: persistent (A) and transient (B) activation. For both groups, the dynamics of Camui activation after the NMDA treatment were not different between somatic and dendritic regions. Therefore, the results indicate that Camui activation dynamic after transient NMDA treatment was uniform in all tested cellular compartments: dendritic spines, dendritic shafts, and cell somas.

### Complex spatial redistribution of Camui in neuronal somas after NMDA treatment

Camui spatial redistribution in neuronal somas after NMDA treatment had multiple phases. During NMDA application, Camui initially changed from almost homogenous distribution to a string-like pattern, with “strings” seeming to be oriented from the nucleus toward the apical dendrite (Fig. 4C, “NMDA”, and S5 Fig., panel D, “NMDA”). Five to ten minutes (7.1 ± 0.4 min on average, n = 7) after the start of the NMDA application, the string-like pattern slowly transformed to numerous small clusters distributed through the whole cell body, as evident in single images taken at different focal planes (S5 Fig., panel C). In several cases, it could be seen that the clusters were also oriented in string-like patterns resembling the pattern formed during the NMDA treatment before clustering (S5 Fig., panel D). These observations suggest that the clusters might be attached to the same intracellular organelles that targeted the Camui during the NMDA treatment. These initial dynamics of Camui redistribution were similar in both groups (with transient and persistent activation, Fig. 4C) except that, in the persistent group, clusters were formed in all cells (100%) shortly after NMDA application and persisted until the end of experiments in ~75% of these cells (Fig. 4B, C, “persistent”), while in the “transient” group, clusters were formed only in 68% of cells and were largely dissolved back into homogenous fluorescence by the end of these experiments (Fig. 4B, C, “transient”). These data seem to suggest that persistence of clusters in cell bodies correlate with the persistence of Camui activation.

### Somatic Camui clusters could persist longer than Camui activation

The correlation between persistence of Camui clustering and persistence of Camui activation (Fig. 4A–C) raised a possibility that the apparent Camui activation state could be a result of Camui self-association when regulatory domains of one holoenzyme bind to and inhibit catalytic domains of neighboring holoenzymes [26,28,29,37,50]. Detailed analysis, however, reveals that clusters in these experiments could persist longer than Camui activation. For example, Fig. 4A shows that at 35 min after NMDA washout, Camui activation completely returned to the baseline while clusters still persisted (Fig. 4C, “transient”). To further investigate this phenomenon, we performed experiments with longer NMDA treatment (7.5 min [Fig. 4D–F] instead of 5 min [Fig. 4A–C]). The longer NMDA exposure not only raised the percentage of cells with persistent Camui activation (65% versus 57%) but also prolonged the duration of the transient activation (~45 min versus ~28 min). Importantly, the longer NMDA application significantly increased the percentage of cells with persistent clusters in the group with transient Camui activation: 71% of cells retained clusters until the end of experiment in comparison to 0% in that group after 5 min NMDA treatment (compare Fig. 4, panels B, E, “transient”). These results further indicate that, similar to what was observed in dendritic regions, the persistence of Camui activation and the stability of its spatial redistribution are not strictly correlated, suggesting that these two processes are regulated by a distinct mechanisms or have different phases.

Changes of Camui fluorescence intensity after NMDA treatment were more complex in cell somas in comparison to that in dendrites (Fig. 4C, F). The somatic fluorescence showed no direct correlation with Camui activation (S8 Fig. and Fig. 4A, D) and could possibly be explained by complex changes in intracellular pH which is known to affect GFP fluorescence [51]. To confirm that NMDA treatment in our experimental conditions also produced clustering of endogenous CaMKII, we preformed immuno-staining of non-transfected cells. S9 Fig. shows that NMDA treatment does cause clustering of endogenous CaMKII.

### Camui mutants I205K and K42R did not prevent cluster formation

Previous studies suggested that CaMKII self-association occurs due to binding of regulatory and catalytic regions of subunits belonging to different holoenzymes [28,29,37]. These studies demonstrated that CaMKII mutations such as I205K (that affects the interaction between CaMKII and NMDA receptor [52]) and K42M (that makes the kinase catalytically dead [53]) prevented CaMKII self-aggregation [28,29,30]. Therefore, we tested whether Camui mutants, I205K or K42R (the latter is another variant of the catalytically dead CaMKII mutant [54,55]) would form clusters in our experimental conditions. S6 Fig. shows that both Camui mutants produced strong persistent activation after the NMDA treatment and formed persistent clusters that lasted until the end of each experiment (~70 min). These results are consistent with the suggestion that persistence of Camui activation and persistence of cluster maintenance are controlled by different mechanisms (more discussion in S4 Text).

### Persistent Camui activity is Ca^2+^- dependent

**Fig 5:**
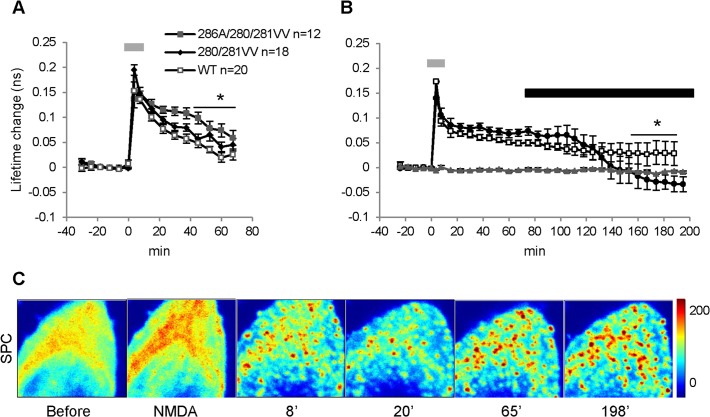
Persistent Camui activity is Ca^2+^-dependent. (A) Summary plots of Camui activation in control experiments (open squares, WT Camui, n = 16) and experiments with Camui mutants (286A/280V/281V, filled squares, n = 18) (280V/281V, n = 12 filled diamond) in response to 7.5 min of the NMDA treatment (grey bar). The magnitude of the activation of Camui mutants was significantly larger than that of WT Camui (thin horizontal line, p < 0.05 for both mutants). (B) Application of ACSF containing 0 mM Ca^2+^ and 5 mM EGTA (black horizontal bar) at 60 min after the 7.5 min of NMDA treatment (grey bar) effectively reversed persistent Camui activation (EGTA, n = 6, circles, p < 0.05), while Camui activity remained persistent in control experiments without lowering Ca^2+^ (Control, n = 6, squares). Baseline control (n = 3, triangles) was stable for the duration of the experiments (> 3.5 hours). (C) SPC images before, during, and at different times after the NMDA treatment show that Camui clusters persisted during the whole experiment and were not dissolved at the end of the experiment after the prolonged exposure to 0 mM Ca^2+^/5 mM EGTA ACSF (198’).

In searching for mechanisms underlying the persistence of Camui activation by NMDA insult, we considered the contribution of T286 autophosphorylation [12,22,23,24,56,57] and C280/M281 oxidation [34,58] to this process: two mechanisms that could lead to persistent CaMKII activity following Ca^2+^/CaM dissociation. To explore the role of oxidation and autophosphorylation, we generated a double mutant that cannot be oxidized on these residues (C280V/M281V) [34], as well as a triple mutant (T286A/C280V/M281V) that is deficient for both the autophosphorylation and the oxidation. Using either of these mutants, we still observed strong persistent activation (Fig. 5A, p < 0.001), indicating that the persistence of Camui activity was not due to autonomous activation by these mechanisms. Interestingly, Camui activation was significantly larger (p < 0.05 for both mutants) than activation of WT Camui, suggesting that both types of autonomous CaMKII states influenced the magnitude of the CaMKII activation in our experimental condition. Clustering was not affected by these mutations: persistent clusters were formed in about 90% of cells in both Camui mutants as well as in WT Camui (S5 Text).

An alternative possible explanation for persistent Camui activation is that it occurred due to a sustained Ca^2+^-dependent process. About 60 min after the NMDA treatments, we replaced standard ACSF with ACSF containing 0 mM Ca^2+^ and 5 mM EGTA to lower extracellular Ca^2+^ level. Fig. 5B shows that, in these experiments, a previously induced persistent Camui activation slowly reverted to the basal level and then below the basal level. In control experiments, in which Ca^2+^ level was not altered, Camui activity remained persistently activated at the level significantly above basal level (0.024 ± 0.012 ns, p < 0.003). A separate control showed that the basal level of Camui activation was stable (-0.008 ± 0.008 ns relative to the period before NMDA, p > 0.05) for the total duration of the experiment (> 3.5 hours). Interestingly, this level was slightly higher than the stable lowest level of Camui activation achieved by prolonged incubation in 0 mM Ca^2+^/EGTA ACSF (-0.32 ns ± 0.015). Although this difference did not reach statistical significance (p = 0.059), there was a strong correlation between basal Camui activity and the depth of the Camui deactivation below the baseline (R^2^ = 0.6, p < 0.05). Together, these latter findings suggest that a population of neurons can have a stable basal level of CaMKII activity and are consistent with the observation that Camui basal activation state in neighboring cells can be different (Fig. 1B). Importantly, although Camui activity during the EGTA treatment decreased below the baseline level, clusters formed after NMDA insult remained stable until the end of all these experiments (> 2.5 hours, n = 6, Fig. 5C). This observation further confirms that the cluster maintenance and the persistent Camui activation are controlled differently.

### NMDA treatment produces persistent mitochondrial damage

**Fig 6:**
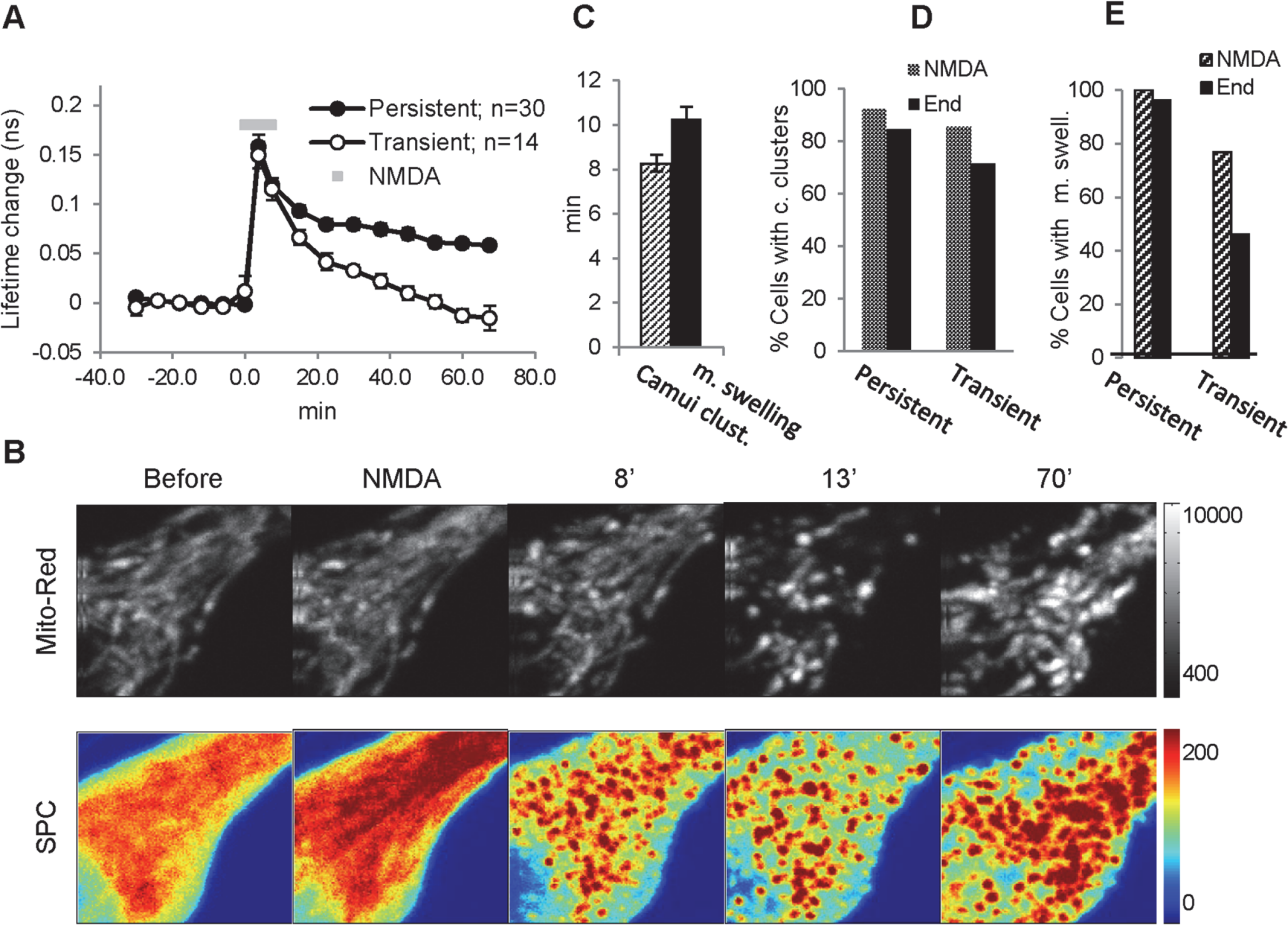
NMDA treatment resulted in a persistent mitochondrial swelling following Camui activation and clustering. (A) Summary graph showing persistent and transient increase in the fluorescence lifetime of Camui after NMDA treatment (grey bar). (B) Representative images of mitochondrial marker (mito-Red) and Camui intensity (SPC) at different times: before, during, and after NMDA application (in minutes). (C) Bar diagram indicating average times for Camui clustering (Camui clust.) and mitochondrial swelling (m. swelling). (D) Bar diagram showing the percentage of cells with CaMKII clustering after the NMDA treatment and at the end of experiments for cells with persistent and transient Camui activation. (E) Bar diagram indicating the percentage of cells with mitochondrial swelling shortly after the NMDA treatment and at the end of experiments for cells with persistent and transient Camui activation.

Ischemia and excitotoxicity are known to produce delayed neuronal cell injury and death [5]. Mitochondrial lesions occur during this period and are thought to be one of the main causes of the ischemic cell injury in both the brain and the heart [39,59]. Therefore, we monitored mitochondrial morphological integrity by expressing fluorescent mitochondrial marker mito-dsRed. The marker clearly labeled mitochondria in somas and dendrites of CA1 neurons without almost any background fluorescence (S7 Fig.). We coexpressed Camui and mito-dsRed and monitored Camui reactivity and mitochondrial integrity simultaneously in neuronal somas before and after the 7.5 min NMDA insult. Under basal conditions, mitochondrial labeling revealed a dense mesh of thin (< 1 μM in diameter) string-like structures filling the cytoplasmic region of the neuronal soma, excluding the nucleus (Fig. 6B, S7 Fig.). NMDA application produced strong Camui activation (Fig. 6A, n = 40) followed by Camui redistribution in a string-like pattern and subsequently in small clusters, as described earlier. Clusters were formed, on average, 8 min (8.3 ± 0.3 min, n = 37) after the start of the NMDA application. At this time, no clear change of mitochondrial morphology was observed (Fig. 6B, 8’). One to five minutes later, however (on average at 10.3 ± 0.4 min, n = 37, after the start of NMDA, Fig. 6C), clear signs of change in mitochondrial morphology were evident: string-like structures seemed to become shorter but larger in diameter (Fig. 6B, 13’, S7 Fig.) [60]. This change in mitochondrial morphology after ischemia has been characterized as swelling [39] although the actual volume could be smaller [61]. The somatic Camui clustering occurred in 100% (n = 27) of experiments in the group of the persistent Camui activation and remained until the end of the experiment in all of these cells (data not shown). The mitochondrial swelling also occurred in all cells of this group, and it persisted until the end of the experiment in 96% of them. In the group with transient Camui activation, clustering occurred in 92% (n = 13) and remained in 85% of them until the end of experiment, while mitochondrial swelling occurred in 77% and remained until the end only in 46% (n = 13) of cells (Fig. 6D, E). Therefore, our protocol for the NMDA-induced toxicity was effective in producing clear signs of prolonged (> 1 hour) mitochondrial damage. Importantly, the magnitude of these toxicity signs correlated with the persistence of Camui activation (Fig. 6E).

## Discussion

The goal of this study was to further investigate the role of CaMKII in neuronal damage induced by ischemic/excitotoxic insults. Specifically, our aim was to complement existing biochemical data with results obtained in living neurons. To achieve this, we employed a high spatial/temporal resolution method of monitoring CaMKII activation and spatial redistribution in different neuronal compartments such as spines, dendritic shafts, and cell somas using CaMKII activity FRET reporter Camui [32,33].

### Activation state of CaMKII in silent neurons at basal conditions

We observed that, under basal conditions in our slice cultures, the Camui activation state was similar in multiple compartments (spine, dendritic shafts, and somas) of individual cells, yet this activation differed considerably between different cells in the same slice. The latter observation was interesting because neuronal firing in our experiments was eliminated by TTX. We hypothesize that the observation could be explained by different cells possessing different resting states of free Ca^2+/^CaM availability, which is not directly coupled to neuronal spiking. Consistent with this idea, the basal Camui activity could be tentatively suppressed below baseline levels by lowering extracellular Ca^2+^ concentration with extracellular EGTA; with the degree of this suppression greatest in cells with higher basal Camui activity. Previous biochemical data demonstrated that the resting level of intracellular Ca^2+^ is sufficient to maintain autonomous activation through persistent Ca^2+^-dependent autophosphorylation at conditions similar to ours (slice cultures, TTX) [62]. Therefore, one explanation of our results could be that some amount of autonomous activity was present at basal conditions in some cells in our experiments as well. However, comparison of fluorescent lifetimes of WT Camui and Camui mutants deficient of autonomous activation showed no significant difference (data not shown), suggesting that the contribution of the autonomously active kinase at basal conditions was minimal or not present at all and that the differences in the basal level of Camui activation may reflect cellular differences in resting cytoplasmic Ca^2+^ /CaM levels (see below).

### Persistent and transient are two major patterns of Camui activation in spines in response to NMDA treatment

We observed two major types of Camui responses to transient (~5–8 min) bath application of NMDA: either persistent or transient activation (group I and group II, respectively). The type of Camui response in different cellular compartments (spines, dendritic shafts, and somas) was very similar, indicating that each cell reacted to the NMDA insult as a single unit. It is not clear what determined the type of Camui reactivity. In most experiments, three cells were imaged simultaneously. Using single-cell electroporation, transfected cells were close to each other and very close to the slice surface (especially somas), minimizing possible differences in local experimental conditions such as ACSF exchange and others. Although the initial peak Camui activation was not different between these neighboring cells, the deactivation rate could be very different. The basal levels of Camui lifetime in spines, dendritic shafts, and cell somas revealed no clear correlation of this parameter with the duration of Camui activation (data not shown). Therefore, some other individual cell characteristics—perhaps different level/activity of endogenous proteins involved in Ca^2+^/CaM homeostasis—might play a role in determining the type of Camui reactivity in a particular cell. When the duration of the NMDA application was increased from 5 to 7.5 min, the rate of Camui deactivation significantly slowed down while the fraction of cells with persistent activation increased. Therefore, the duration of a focal ischemia-induced excitotoxic insult *in vivo* might play a role in determining the overall duration of CaMKII activation in affected neurons.

### CaMKII redistribution in dendrites and spines

Camui activation by NMDA treatment often coincided with an increase of Camui amount in spines and its decrease in dendrites. This observation is consistent with previous cell biological studies showing that strong cell excitation or ischemia resulted in translocation of CaMKII to spines or synaptic puncta [28,41,48]. Ultrastructural EM studies of neurons following excitotoxic calcium signaling revealed that CaMKII accumulates in the postsynaptic density (PSD) of excitatory synapses [41,49]. Some of the kinase that translocates to spines may exist in multimeric structures, similar to self-associated aggregates that form within the cytoplasm following strong excitation or ischemia [26,28,29,37,63]. Although the duration of the dendritic/spine redistribution of Camui amount observed in our experiments, in general, correlated with the duration of Camui activation, often the duration of Camui redistribution into spines lasted longer than Camui activation (Figs. 2, 3 and S2 Fig.). This suggests that, unlike the initiation of the CaMKII redistribution to spines, which requires CaMKII activation, the maintenance of the translocated kinase may not need CaMKII activation, indicating different regulation or phases of these two processes (see additional discussion below).

### CaMKII activation and clustering in cell somas

In cell bodies, Camui distribution after NMDA treatment underwent a complex spatial transformation. The initial transformation of near homogeneous Camui distribution into a string-like pattern resembles activity-dependent translocation of CaMKII from cytosol to microtubules [55]. Similarly to the microtubule translocation, the string-like pattern that we observed was very short-lasting (1–3 min), being quickly replaced by numerous dense clusters. These clusters closely resembled large (~100–300 nm) globular self-associated CaMKII aggregates/clusters described by others in primary cells at ischemic/excitotoxic conditions [26,28,29,63]. In *in vitro* experiments, CaMKII cluster formation required Ca^2+^/CaM-dependent transition of CaMKII into open “active” conformation, whereby aggregated CaMKII molecules slowly lost their Ca^2+^/CaM-dependent and-independent activity toward peptide substrates [50]. The formation of these clusters, termed self-association, was proposed to result from the regulatory and catalytic domains of different CaMKII holoenzymes forming inter-holoenzyme binding [28,29,37]—a process that locks associated subunits in an open but enzymatically inactive state. Thus, in a simplified model, most Camui molecules could maintain persistent open conformation (“activation”) observed in our study due to this type of molecular association. Further analysis, however, suggested against this simple model: Camui could change its conformation state from open “active” to closed “inactive” conformation without dramatic change in its clustering level. One possibility is that the mechanism of cluster maintenance in our experimental conditions was different from that described in the previous publications. Consistent with this possibility, two Camui variants, with mutations that prevented CaMKII clustering in previous studies (I205K and K42R) [28,29,30], did not alter clustering in our experiments (see S4 Text for additional discussion). Another interpretation is that, in our experiments, Camui formed loosely packed clusters, with only a few subunits in each dodecameric holoenzyme participating in the self-association type aggregating reaction. In this case, all other subunits would retain their ability to change conformation from open (active) to closed (inactive) state without affecting cluster structure. Consistently, these loosely packed clusters of partially active CaMKII were observed *in vitro* during early phases of the kinase aggregation [37,50]. Regardless of the cluster maintenance mechanism, the clustered kinase, while retaining ability for activation, is likely to have restricted enzymatic function due to a loss of its proper targeting and substrate availability, as previously postulated [26,37]. Therefore, our data suggest that, in a large population of cells in our experiments, excitotoxic insult produced a persistent Ca^2+^-dependent activation of CaMKII, with restricted function due to strong kinase clustering.

### Contribution of Ca^2+^-dependent and autonomous states in persistent CaMKII activation

Our analysis suggest that the persistent Camui activation after the NMDA treatment was mostly Ca^2+^-dependent due to a prolonged elevation of cytosolic calcium, with a little impact of autonomous state. This conclusion is based on two observations: first, the persistent Camui reactivity was not blocked by CaMKII mutations, which prevent generation of autonomous states through either autophosphorylation of T286 or oxidation of C280/M281; second, the persistent Camui activation was reversed when Ca^2+^ was replaced by EGTA in the bath ACSF. These observations, however, do not exclude that a fraction of CaMKII pool was either autophosphorylated at T286 or oxidized at C280/M281 because these modifications seem to contribute very little if any to the total Ca^2+^/CaM-stimulated activity of the kinase [9,34,58,64,65]. Consistently, we found that the magnitude of the persistent Camui activation was only marginally influenced by mutations preventing autonomous (“open”) states of CaMKII trough T286 autophosphorylation or C280/M281 oxidation. Biochemical works *in vitro* reported that T286 phosphorylation could either increase or decrease the total Ca^2+^-stimulated CaMKII activity, indicating that results could depend significantly on experimental conditions ([64,65] and others). Our data suggest that in the environment of the living cell, the autonomous activation slightly decreases the total Ca^2+^-stimulated WT CaMKII activation because the autonomous-deficient CaMKII mutants used in our study produced slightly larger persistent activation. Independent of the interpretation, the results suggest that some CaMKII autonomous state did occur after NMDA challenge. Importantly, our study also revealed that Camui mutants that are deficient exclusively in oxidation-dependent autonomous state (CM28/281VV) also showed significantly larger persistent activation than that of WT Camui. This finding strongly indicates that this specific type of oxidation-dependent CaMKII regulation can be activated in neurons at excitotoxic conditions. To our knowledge, our results are the first demonstration of the existence of this type of oxidation-dependent CaMKII regulation in the brain confirmed by the mutational analysis.

### Is CaMKII autonomous state involved in excitotoxic cell damage?

In ischemic heart, CaMKII autonomous activation has been suggested to play a critical role in promoting cell damage, though the mechanisms are not completely understood [66,67,68]. Recent studies also implicated the autonomous state of CaMKII in neuronal damage produced by ischemia/excitotoxicity [2,4], but that mechanisms are also not clear. Our finding that autonomous state of CaMKII was generated in neurons by an excitotoxic insult in general is consistent with this implication. It seems puzzling, however, how the T286 autophosphorylated kinase state, which contributes very little if at all to the total Ca^2+^-stimulated enzymatic activity [9,64,65], can cause a significant effect on cell toxicity? Two possibilities look plausible. One is that cell Ca^2+^ was not persistently elevated during the post-stroke period in the experimental conditions of that study. Another intriguing possibility is that CaMKII participated in toxic processes not through its enzymatic autonomous activity but through targeting which requires its T286 autophosphorylation even in the presence of elevated Ca^2+^. CaMKII binding to NR2B receptor is one form of this type of targeting [52] and it was shown to play a role in ischemic toxicity [24,69]. Notably, CN21 peptide inhibitor of CaMKII which inhibits post-stroke cell toxicity [2,3] not only interferes with formation of CaMKII/NMDAR complex *in vitro* [70] but also reduces the existing complex in living brain slices [71]. Our finding that most of Camui activation after the excitotoxic insult was Ca^2+^-dependent with a little impact of autonomous CaMKII state is in agreement with this last interpretation. It would be interesting to implement FRET based imaging of CaMKII targeting after excitotoxic insults to test this hypothesis.

### Persistent Ca^2+^ elevation, CaMKII activation and mitochondrial damage

Although cytoplasmic Ca^2+^ overload in general is the integral feature of ischemia/reperfusion processes, it is the long-lasting Ca^2+^ elevation that has been linked to delayed cell death after ischemia [39,59]. A prolonged (a few minutes to many hours) Ca^2+^ rise in neurons after short insults was reported both in animals *in vivo* and in live neurons *in vitro* [72,73,74,75]. Mitochondrial Ca^2+^ overload and following mitochondrial damage has been considered as one of the most likely causes of this sustained Ca^2+^ increase (delayed Ca^2+^ deregulation) and cell injury [39,59]. If the initial Ca^2+^ overload does not exceed critical level, the mitochondrial damage may recover, leading to restoration of intracellular Ca^2+^ and to cell survival [39,59]. Our data seems to be in line with this hypothesis. We found that mitochondrial swelling occurred with a delay of several minutes after NMDA insult and the persistence of this mitochondrial damage correlates with the persistence of Camui activation which is mostly Ca^2+^-dependent. Therefore, it is plausible that the persistence of CaMKII activation after excitotoxic insult is controlled by Ca^2+^ deregulation, which at least in part depends on mitochondrial damage. In addition, over-activated CaMKII itself has a potential to promote the toxic deregulation of Ca^2+^ homeostasis [66,67,68,76].

## Conclusions

Our results support the idea that an excitotoxic insult produces a persistent Ca^2+^-dependent activation of CaMKII, with a limited impact of the kinase autonomous states dependent on T286 phosphorylation or C280/M281 oxidation. The overall functional enzymatic effect of the kinase, however, is likely to be restricted due to its strong clustering. Further studies are needed to elucidate a mechanism of the involvement of CaMKII in neuronal excitotoxicity.

## Supporting Information

S1 Fig Negligible level of correlation between Camui activity (fluorescence lifetime) and its content (fluorescence intensity) in neuronal cell bodies or dendritic spines. Each data point represents the number of photons (fluorescence intensity) and the average fluorescence lifetime for each cell soma (A) or spine (B). Data were normalized to the average fluorescence intensity and fluorescence lifetime for all cells or spines in each image or branch, respectively.
(TIF)

S2 Fig Two examples showing transient Camui activation but persistent increase in its spine content after NMDA treatment. (A) Plots of Camui fluorescence lifetime (top panels) showing that application of NMDA (grey bar) produced only a transient increase of the lifetime, but a persistent increase in Camui fluorescence in spines (bottom panels). (B) FLIM and SPC images before, during NMDA application, and 34 min of washout for the experiment shown in (A). Scale bar, 1 μm.
(TIF)

S3 Fig Additional types of Camui reactivity (groups III and IV) have different characteristics. Groups are characterized by noisy or no lifetime response (A), dramatic decrease of Camui fluorescence in dendrite (B) and concomitant dendritic swelling (C). See S1 Table.
(TIF)

S4 Fig Morphological transformations produced by NMDA treatment: spine growth, collapse, and reappearance. (A-C) SPC images taken during different times in three experiments showing slight swelling/shrinkage (A, B, asterisk), an increase in spine fluorescence intensity (A, B, arrows), and appearance of new spines (B, C, arrows) induced by NMDA application; these and other spines disappeared at later times after the NMDA washout. (D) Images show appearance of filopodia (arrow) at 32 min after the NMDA treatment, but most spines were collapsed during the later time of the washout (80 min). (E) Many spines collapsed 15 min after the first NMDA treatment, but some reappeared (arrows) after the second treatment.
(TIF)

S5 Fig NMDA treatment resulted in the formation of small dense clusters of Camui through the whole volume of cell body. Camui reactivity in cell bodies (open squares) and dendrites (open circles) of the same cell was similar: (A)—persistent, (B)—transient. Note that, in these experiments, dendritic regions were not imaged during the NMDA treatment, and therefore the comparison can be made only for the period after the treatment when imaging of both regions was done alternatively. (C) Two columns show individual consecutive Z-axis frames from two SPC image stacks taken during imaging of two different cell bodies. Numbers on the left indicate frame numbers starting from cell surface and proceeding deeper inside the cell with steps of 1 μm; it is evident that new clusters appear and disappear every 1–2 Z steps. Imaging the same clusters in two consecutive Z steps is expected because the Point Spread Function in Z axis at this wavelength is ~2.6 μm. (D) SPC projection images during NMDA treatment (left) before cluster formation and 7 min after NMDA treatment when clusters were formed (right). Note similar “string-like” pattern of Camui distribution in both images (arrows).
(TIF)

S6 Fig Camui mutants I205K and K42R did not prevent cluster formation. (A) Summary plot of fluorescent lifetime change showing that both Camui mutants (I205K, triangles and K42R, squares) produced persistent activation after 7.5 min NMDA treatments. (B) Representative images of Camui fluorescence (SPC) for each mutant, demonstrating that clusters formed shortly after the NMDA treatment (NMDA) persisted until the end of experiments ~70 min (70’). (C) Summary plot showing that fraction of cells that formed clusters shortly after NDMA treatment (NMDA) and the fraction that retained these clusters until the end of experiments (END) were not significantly different between Camui mutants (I205K and K42R) and control experiments (WT).
(TIF)

S7 Fig A representative example of mito-DsRed expression in a CA1 neuron. (A) A low-magnification image of a CA1 neuron showing strong mito-DsRed expression in cell soma and both apical and basal dendrites. (B–D) Higher-magnification images showing mitochondrial network in cell body (B), a segment of proximal dendrite (C), and a more distal segment of apical dendrite (D). (E), (F) Somatic mitochondrial network in control conditions. (E) Two images at times 5 min and 0 min before NMDA and after NMDA treatment. (F) Two images at 10 and 50 min of washout; note clear mitochondrial swelling produced by the NMDA treatment.
(TIF)

S8 Fig Change in Camui content in cell bodies after 5 (A) and 7.5 (B) min of NMDA treatment. The data are complementary to Fig. 4. During NMDA application, there was always a short period of increase in Camui content in cell bodies. At ~5–10 min after the NMDA application, the Camui intensity in cell bodies reverted to the baseline level. The Camui fluorescence intensity in the “persistent” group (open symbols) remained near the baseline during the ~ 20–70 minute period, while in the “transient group” (filled symbols), Camui fluorescence increased again after 20 min and remained elevated until the end of the experiment.
(TIF)

S9 Fig Immunostaining of endogenous CaMKII in untransfected cells shows CaMKII clustering after the NMDA insult. Experiment: Slices with cells coexpressing Camui and mito-dsRed were treated with NMDA (25μM) and then fixed and processed for CaMKIIα immunostaining. (A) Camui lifetime change produced by NMDA treatment (average of 4 cells, arrow indicates the time of slice fixation). (B) Images of one of cells from the experiment shown in (A) showing mito-dsRed fluorescence before (control) and after NMDA treatment (NMDA): note mitochondrial swelling produced by NMDA. (C) Images of one of cells from the experiment shown in (A) showing pattern of Camui distribution (GFP fluorescence) before (control) and after NMDA treatment (NMDA): note Camui clustering. (D) Images of CaMKIIα immunostaning (Alexa 595 tagged secondary antibody) of cells in experiment shown in (A); insert in the right, top corner shows higher magnification of the region indicated in the left bottom side of the image; note the endogenous CaMKII clustering after NMDA treatment.
(TIF)

S1 Table Characteristics of Camui reactivity and morphological changes in different neuronal populations. (DOCX)

S1 Text Camui activity state and content distribution at basal conditions. (DOCX)

S2 Text Morphological transformations produced by NMDA treatment: spines growth, collapse, and reappearance. (DOCX)

S3 Text Statistical data for summary results shown in Fig. 3. (DOCX)

S4 Text Camui mutants I205K and K42R did not prevent cluster formation. (DOCX)

S5 Text Clustering was not affected by mutations that prevented CaMKII autonomous state by either autophosphorylation of T286 or oxidation of C280/M281. (DOCX)
