# Supplementary figures and images for: Excitotoxic Insult Results in a Long-Lasting Activation of CaMKIIα and Mitochondrial Damage in Living Hippocampal Neurons
Source: PLoS One. 2015 Mar 20;10(3):e0120881. doi: 10.1371/journal.pone.0120881 (PMC4368532; doi:10.1371/journal.pone.0120881)

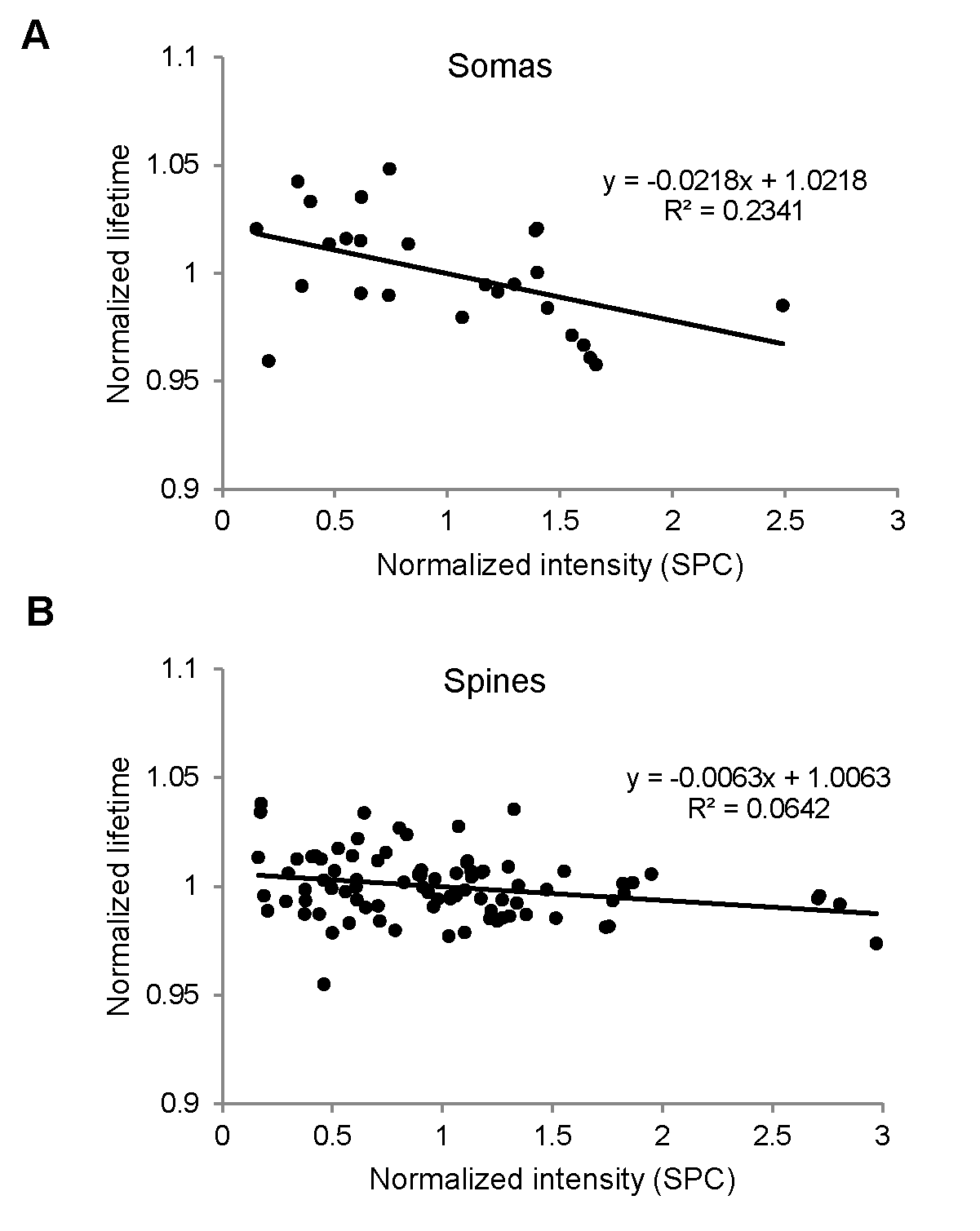

Supplement: S1 Fig — Each data point represents the number of photons (fluorescence intensity) and the average fluorescence lifetime for each cell soma (A) or spine (B). Data were normalized to the average fluorescence intensity and fluorescence lifetime for all cells or spines in each image or branch, respectively. (TIF) [file pone.0120881.s001.tif]

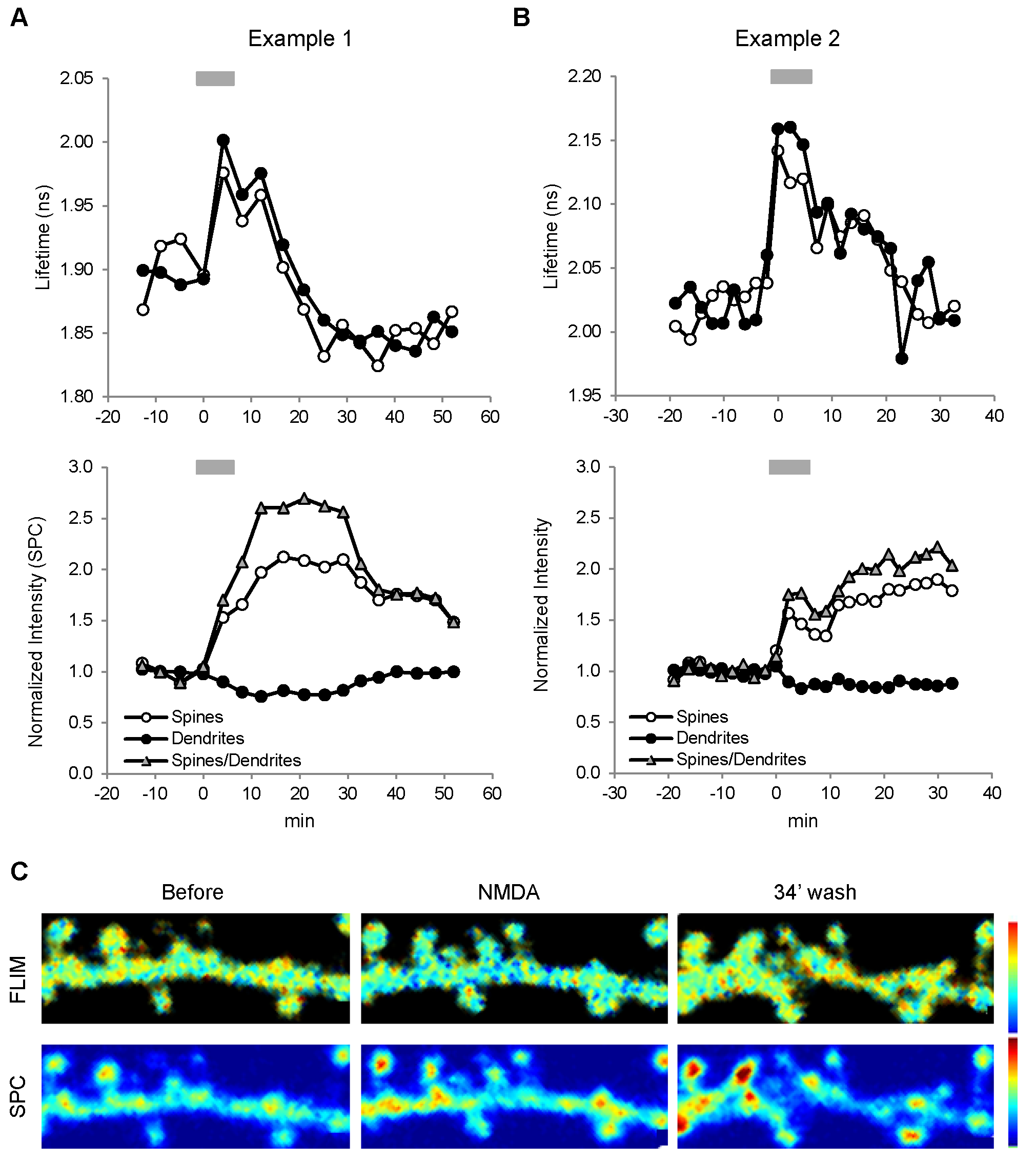

Supplement: S2 Fig — (A) Plots of Camui fluorescence lifetime (top panels) showing that application of NMDA (grey bar) produced only a transient increase of the lifetime, but a persistent increase in Camui fluorescence in spines (bottom panels). (B) FLIM and SPC images before, during NMDA application, and 34 min of washout for the experiment shown in (A). Scale bar, 1 μm. (TIF) [file pone.0120881.s002.tif]

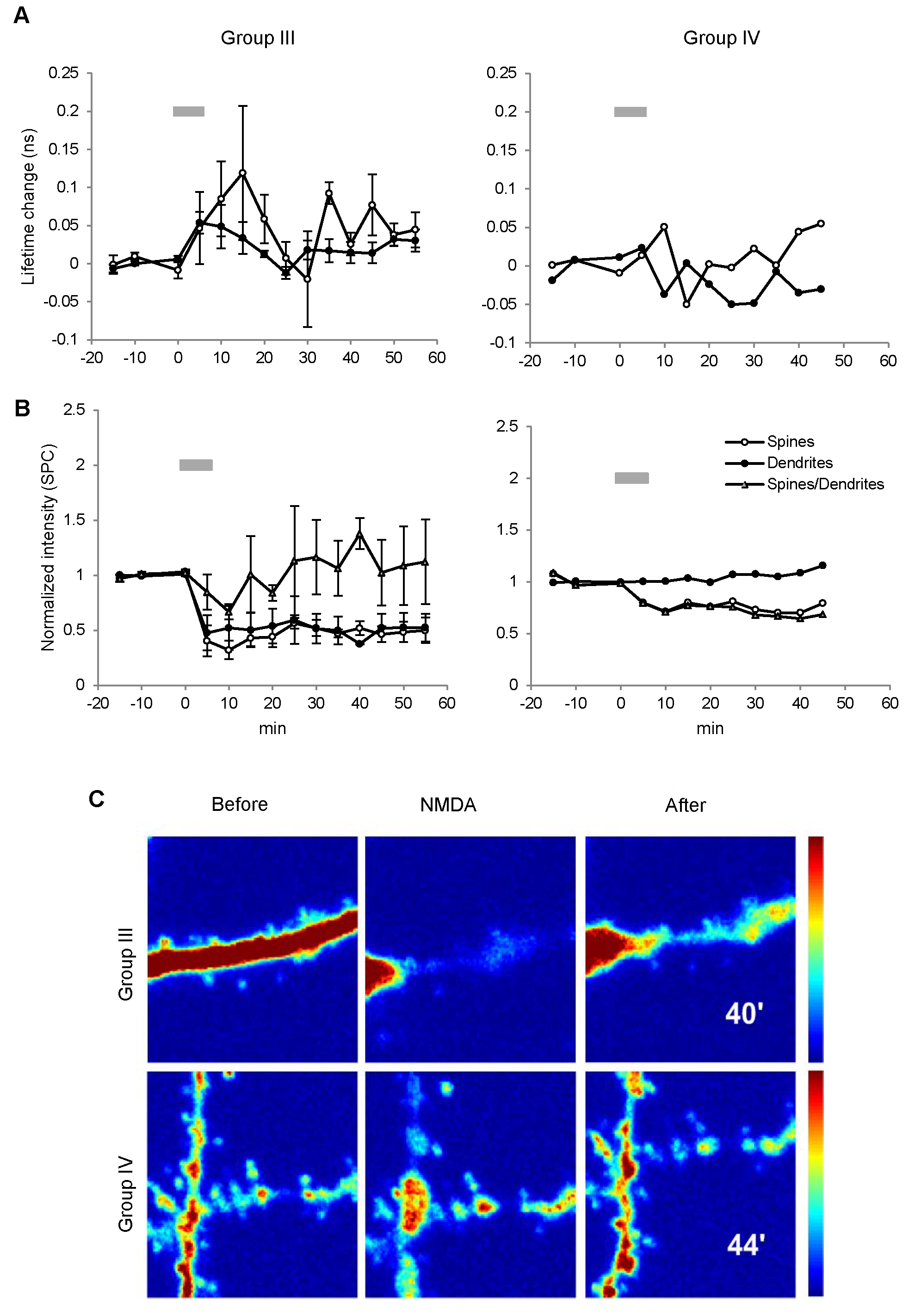

Supplement: S3 Fig — Groups are characterized by noisy or no lifetime response (A), dramatic decrease of Camui fluorescence in dendrite (B) and concomitant dendritic swelling (C). See S1 Table. (TIF) [file pone.0120881.s003.tif]

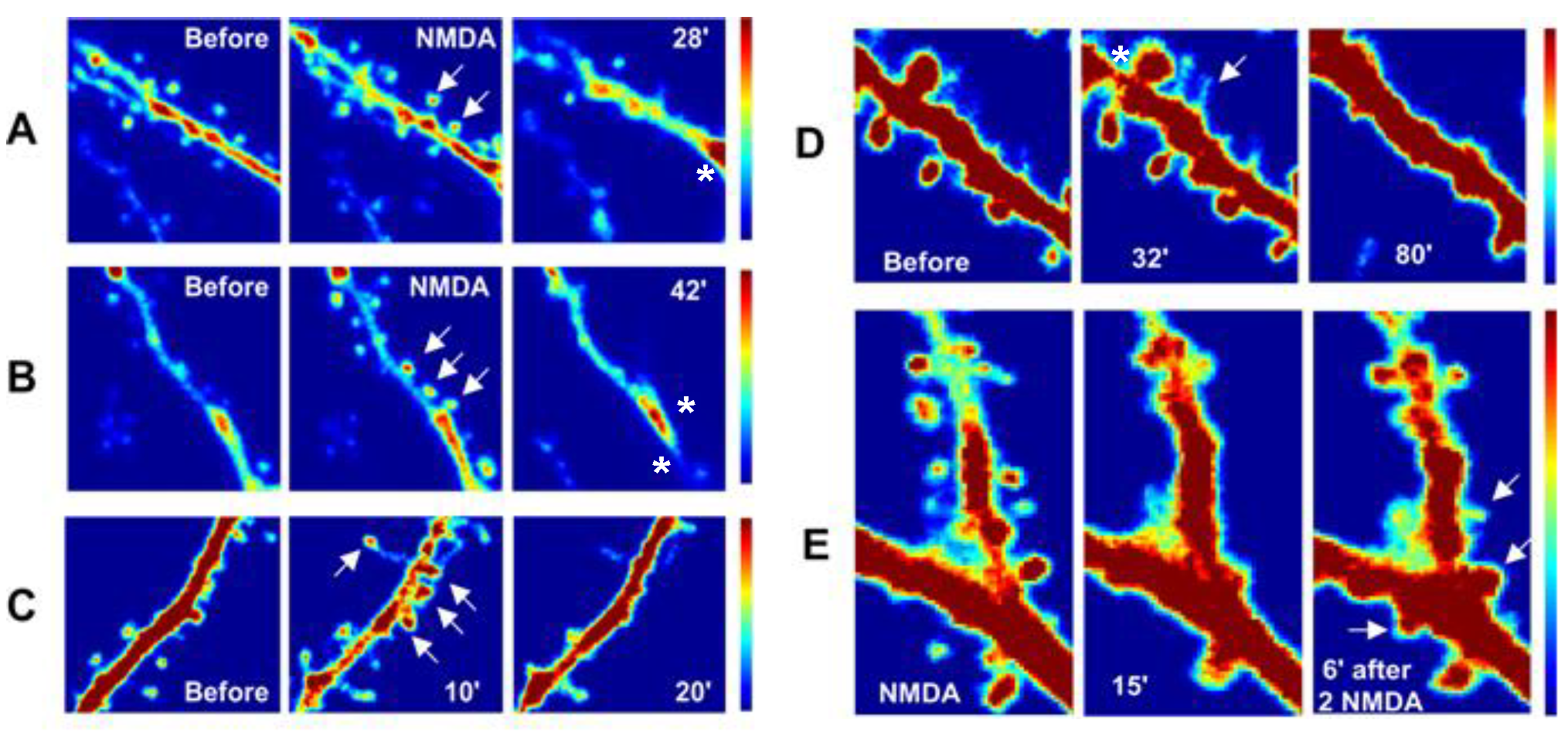

Supplement: S4 Fig — (A-C) SPC images taken during different times in three experiments showing slight swelling/shrinkage (A, B, asterisk), an increase in spine fluorescence intensity (A, B, arrows), and appearance of new spines (B, C, arrows) induced by NMDA application; these and other spines disappeared at later times after the NMDA washout. (D) Images show appearance of filopodia (arrow) at 32 min after the NMDA treatment, but most spines were collapsed during the later time of the washout (80 min). (E) Many spines collapsed 15 min after the first NMDA treatment, but some reappeared (arrows) after the second treatment. (TIF) [file pone.0120881.s004.tif]

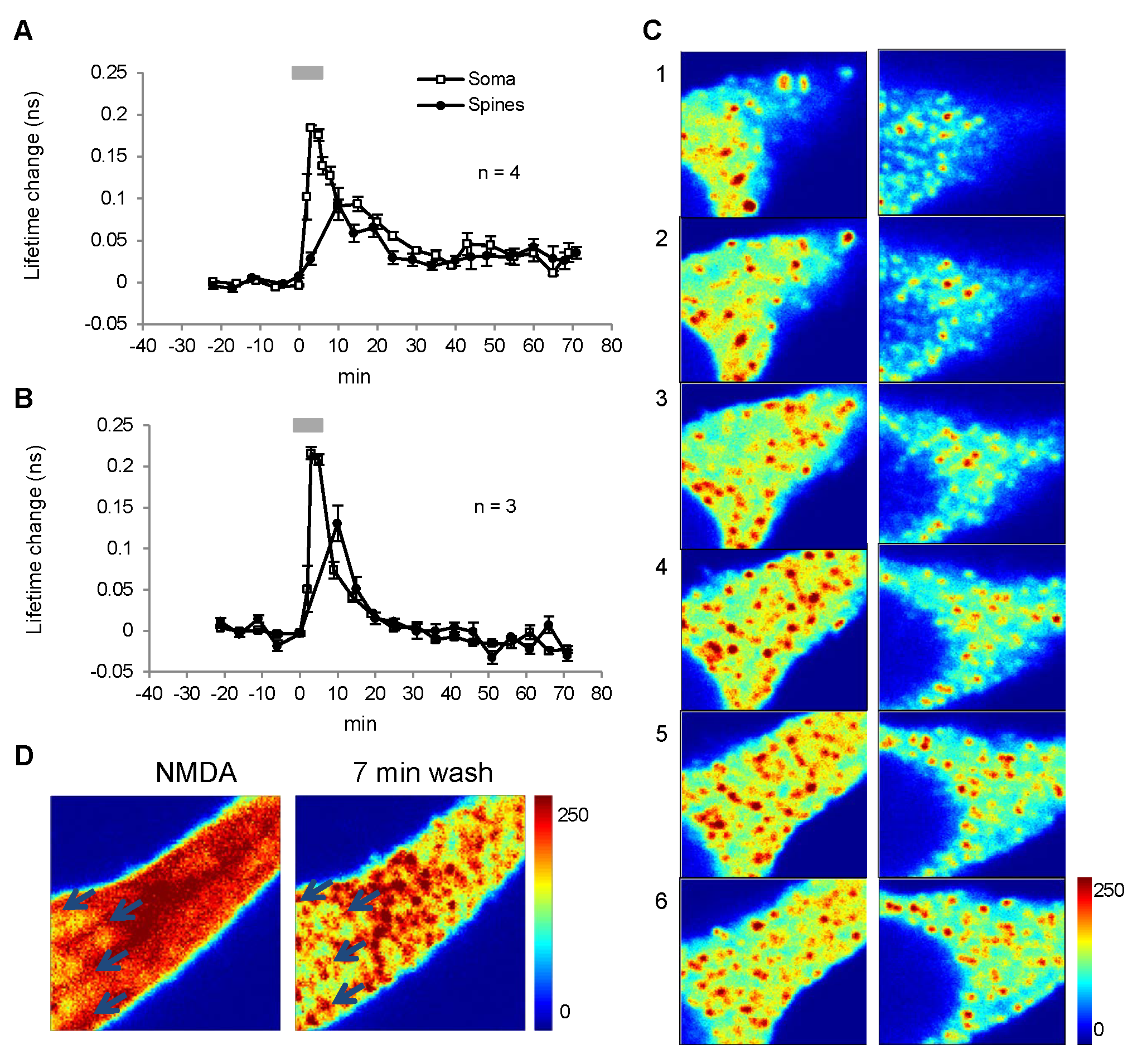

Supplement: S5 Fig — Camui reactivity in cell bodies (open squares) and dendrites (open circles) of the same cell was similar: (A)—persistent, (B)—transient. Note that, in these experiments, dendritic regions were not imaged during the NMDA treatment, and therefore the comparison can be made only for the period after the treatment when imaging of both regions was done alternatively. (C) Two columns show individual consecutive Z-axis frames from two SPC image stacks taken during imaging of two different cell bodies. Numbers on the left indicate frame numbers starting from cell surface and proceeding deeper inside the cell with steps of 1 μm; it is evident that new clusters appear and disappear every 1–2 Z steps. Imaging the same clusters in two consecutive Z steps is expected because the Point Spread Function in Z axis at this wavelength is ~2.6 μm. (D) SPC projection images during NMDA treatment (left) before cluster formation and 7 min after NMDA treatment when clusters were formed (right). Note similar “string-like” pattern of Camui distribution in both images (arrows). (TIF) [file pone.0120881.s005.tif]

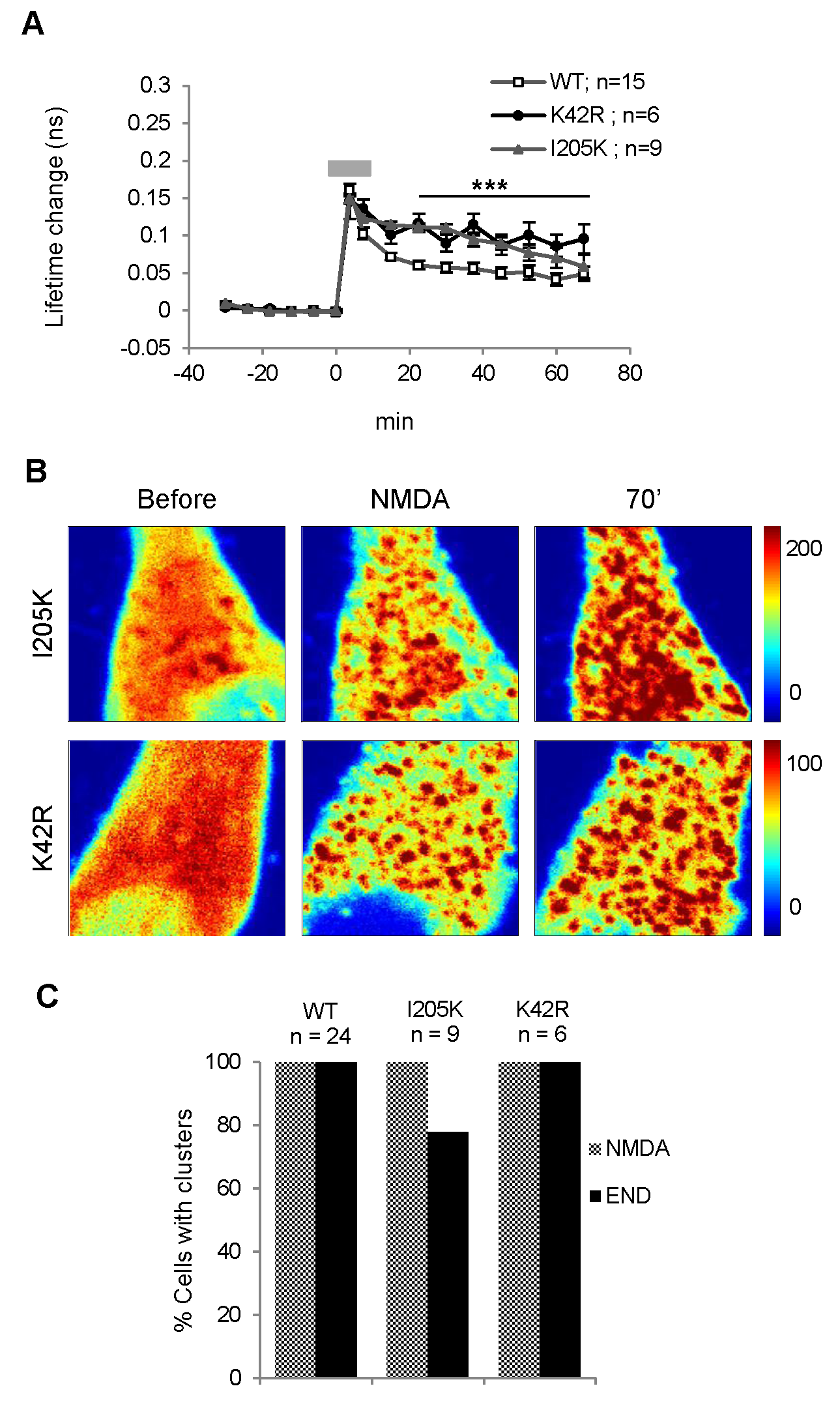

Supplement: S6 Fig — (A) Summary plot of fluorescent lifetime change showing that both Camui mutants (I205K, triangles and K42R, squares) produced persistent activation after 7.5 min NMDA treatments. (B) Representative images of Camui fluorescence (SPC) for each mutant, demonstrating that clusters formed shortly after the NMDA treatment (NMDA) persisted until the end of experiments ~70 min (70’). (C) Summary plot showing that fraction of cells that formed clusters shortly after NDMA treatment (NMDA) and the fraction that retained these clusters until the end of experiments (END) were not significantly different between Camui mutants (I205K and K42R) and control experiments (WT). (TIF) [file pone.0120881.s006.tif]

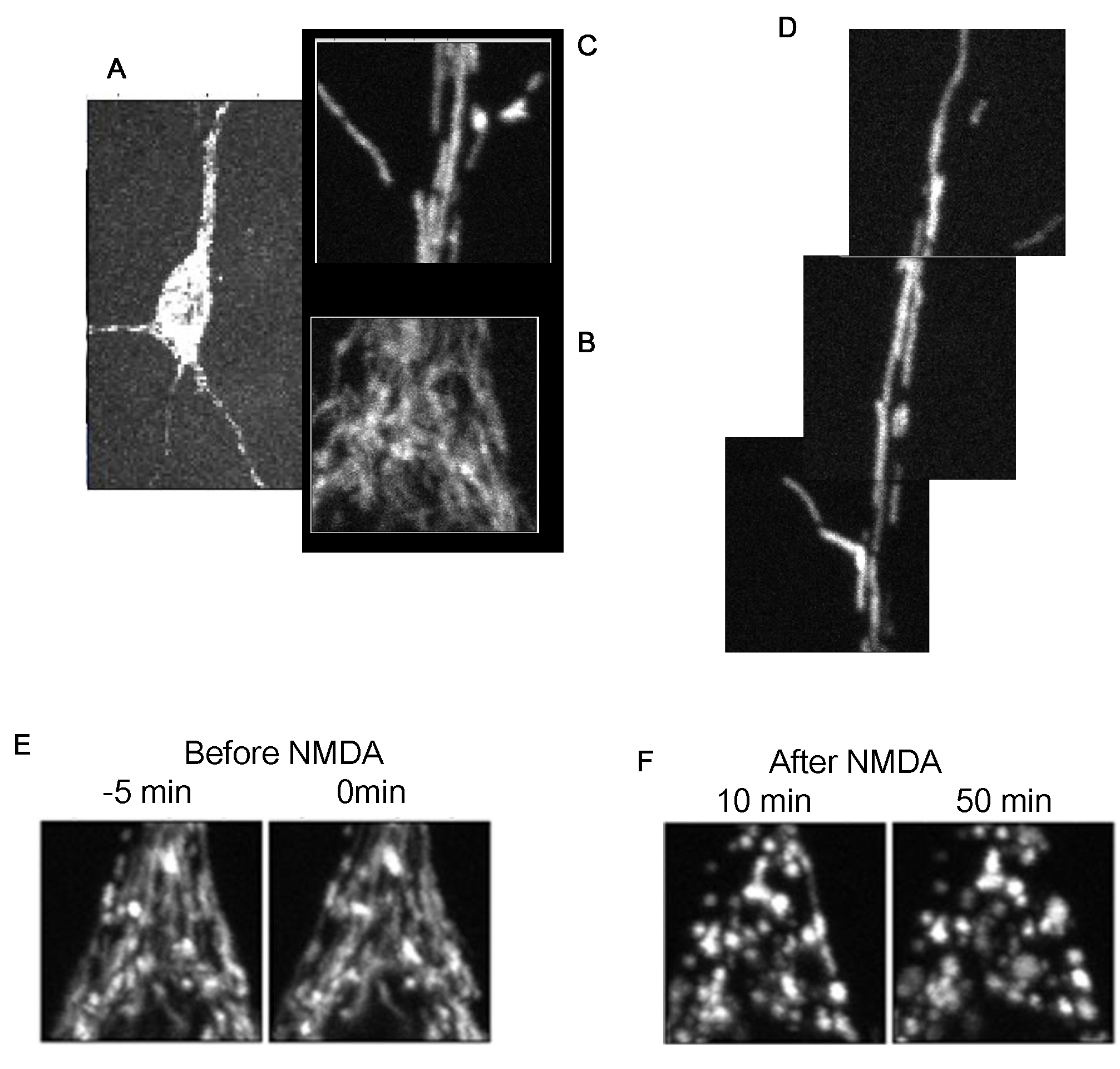

Supplement: S7 Fig — (A) A low-magnification image of a CA1 neuron showing strong mito-DsRed expression in cell soma and both apical and basal dendrites. (B–D) Higher-magnification images showing mitochondrial network in cell body (B), a segment of proximal dendrite (C), and a more distal segment of apical dendrite (D). (E), (F) Somatic mitochondrial network in control conditions. (E) Two images at times 5 min and 0 min before NMDA and after NMDA treatment. (F) Two images at 10 and 50 min of washout; note clear mitochondrial swelling produced by the NMDA treatment. (TIF) [file pone.0120881.s007.tif]

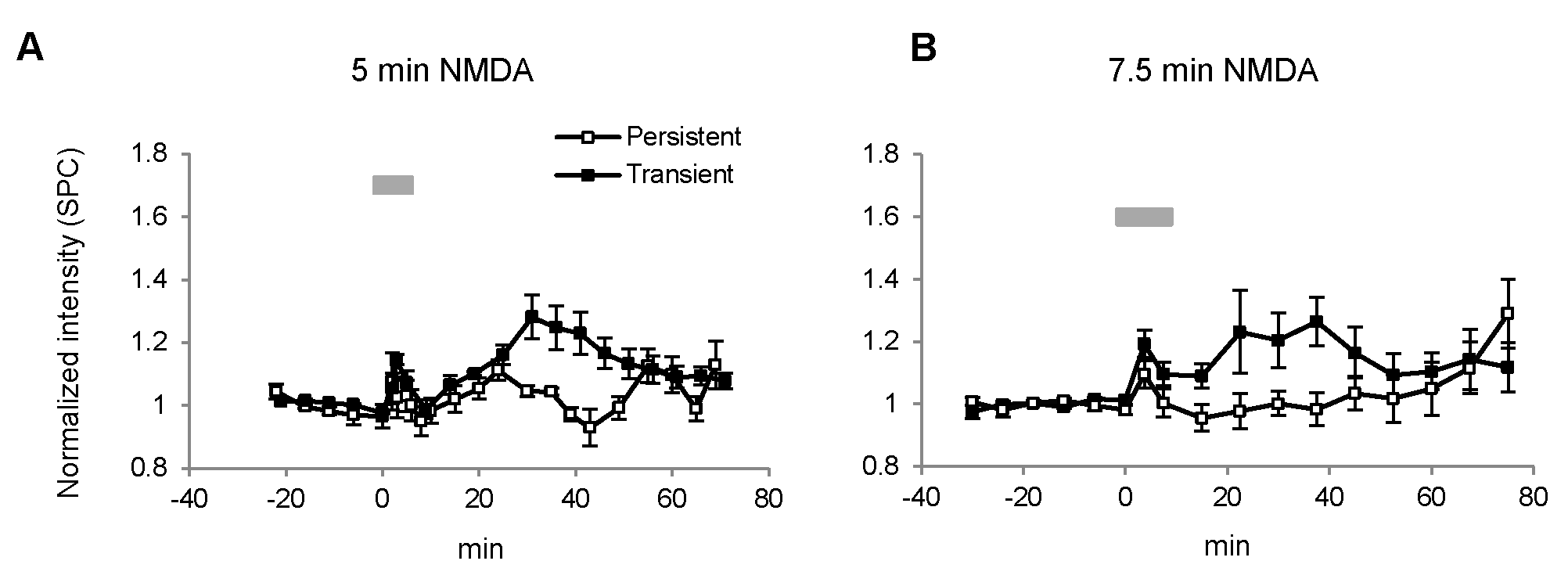

Supplement: S8 Fig — The data are complementary to Fig. 4. During NMDA application, there was always a short period of increase in Camui content in cell bodies. At ~5–10 min after the NMDA application, the Camui intensity in cell bodies reverted to the baseline level. The Camui fluorescence intensity in the “persistent” group (open symbols) remained near the baseline during the ~ 20–70 minute period, while in the “transient group” (filled symbols), Camui fluorescence increased again after 20 min and remained elevated until the end of the experiment. (TIF) [file pone.0120881.s008.tif]

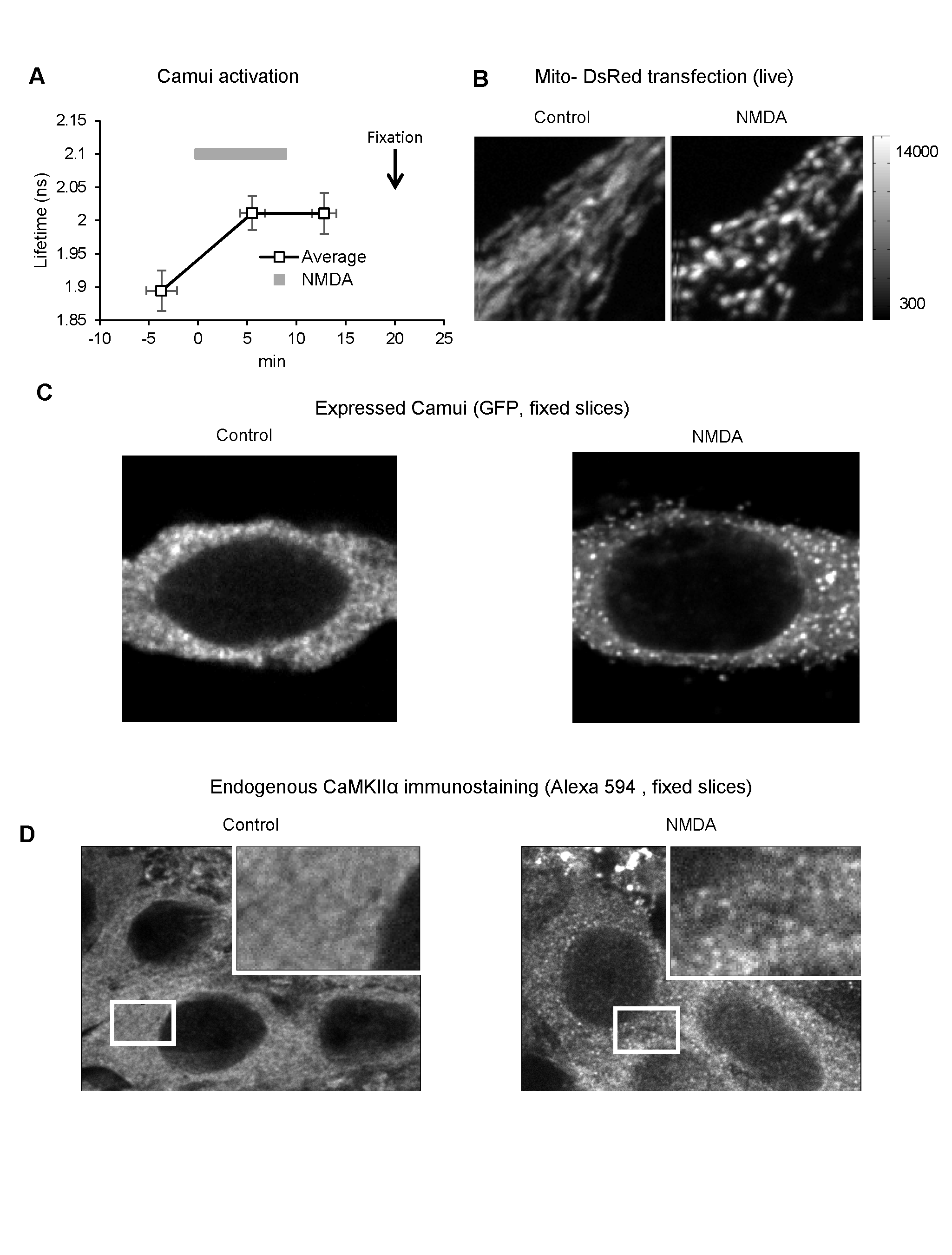

Supplement: S9 Fig — Experiment: Slices with cells coexpressing Camui and mito-dsRed were treated with NMDA (25μM) and then fixed and processed for CaMKIIα immunostaining. (A) Camui lifetime change produced by NMDA treatment (average of 4 cells, arrow indicates the time of slice fixation). (B) Images of one of cells from the experiment shown in (A) showing mito-dsRed fluorescence before (control) and after NMDA treatment (NMDA): note mitochondrial swelling produced by NMDA. (C) Images of one of cells from the experiment shown in (A) showing pattern of Camui distribution (GFP fluorescence) before (control) and after NMDA treatment (NMDA): note Camui clustering. (D) Images of CaMKIIα immunostaning (Alexa 595 tagged secondary antibody) of cells in experiment shown in (A); insert in the right, top corner shows higher magnification of the region indicated in the left bottom side of the image; note the endogenous CaMKII clustering after NMDA treatment. (TIF) [file pone.0120881.s009.tif]
