# Supplementary material for: Excitotoxic Insult Results in a Long-Lasting Activation of CaMKIIα and Mitochondrial Damage in Living Hippocampal Neurons
Source: PLoS One. 2015 Mar 20;10(3):e0120881. doi: 10.1371/journal.pone.0120881 (PMC4368532; doi:10.1371/journal.pone.0120881)
Supplement: S1 Table — (DOCX) [file pone.0120881.s010.docx]

**S1 Table. Characteristics of Camui reactivity and morphological changes in different neuronal populations**

|  | **Camui activation** | **Camui redistribution** | **Dendritic swelling/shrinkage** |
| --- | --- | --- | --- |
| **Group I (7/16)** | Persistent | Persistent **increase** in spines and dendrites | Small |
| **Group II (5/16)** | Transient | Transient **increase** in spines and dendrites | Small |
| **Group III (3/16)** | Noisy | Persistent **decrease** in spines and dendrites | Strong |
| **Group IV (1/6)** | No change | Persistent **decrease** in spines; no change in dendrites | Strong |
