# Supplementary material for: Excitotoxic Insult Results in a Long-Lasting Activation of CaMKIIα and Mitochondrial Damage in Living Hippocampal Neurons
Source: PLoS One. 2015 Mar 20;10(3):e0120881. doi: 10.1371/journal.pone.0120881 (PMC4368532; doi:10.1371/journal.pone.0120881)
Supplement: S1 Text — (DOCX) [file pone.0120881.s011.docx]

**S1 Text. Camui activity state and content distribution at basal conditions**

After 30 min of pre-incubation, the fluorescent lifetime in spines and dendrites was stable for a 20–30 min period of baseline recording before NMDA application (at -20 min, fluorescent lifetime was 2.00 ± 0.03 ns in spines and 1.98 ± 0.03 ns in dendrites, in comparison to 1.98 ± 0.03 ns in spines and 1.97 ± 0.03 ns in dendrites at 0 min, p > 0.05, n = 27).

The large variance of fluorescence lifetime in neighboring cells could not be explained by recording noise, which was below 0.003 ns, nor by the variability in the level of expression of Camui, as the correlation between the level of Camui expression in different cells and its fluorescent lifetime was negligible (n = 26, R^2^ = 0.23; p < 0.05**,** S1 Fig., panel A). The level of Camui activation showed no positive correlation with the spine content of Camui (S1 Fig., panel B). Other studies suggested that the synaptic accumulation of the kinase was promoted by CaMKII autonomous activation [[1](#_ENREF_1)], possibly, through binding to synaptic NMDA receptors while being in an active (open) state [[2](#_ENREF_2),[3](#_ENREF_3)]. Therefore, together with finding that the amount of CaMKII in spines correlates with spine size and synaptic strength [[4](#_ENREF_4)], one could expect a positive correlation of the kinase activation state and its spine content. The fraction of the CaMKII subunits that are directly bound to the NMDA receptors at basal conditions, however, is very small [[5](#_ENREF_5)], and therefore unlikely to be detected using the current methodology of fluorescence lifetime measurements.

1. Bayer KU, LeBel E, McDonald GL, O'Leary H, Schulman H, et al. (2006) Transition from reversible to persistent binding of CaMKII to postsynaptic sites and NR2B. J Neurosci 26: 1164-1174.

2. Bayer KU, De Koninck P, Leonard AS, Hell JW, Schulman H (2001) Interaction with the NMDA receptor locks CaMKII in an active conformation. Nature 411: 801-805.

3. Colbran RJ (2004) Targeting of calcium/calmodulin-dependent protein kinase II. Biochem J 378: 1-16.

4. Asrican B, Lisman J, Otmakhov N (2007) Synaptic strength of individual spines correlates with bound Ca2+-calmodulin-dependent kinase II. J Neurosci 27: 14007-14011.

5. Feng B, Raghavachari S, Lisman J (2011) Quantitative estimates of the cytoplasmic, PSD, and NMDAR-bound pools of CaMKII in dendritic spines. Brain Res.
