# Supplementary material for: Excitotoxic Insult Results in a Long-Lasting Activation of CaMKIIα and Mitochondrial Damage in Living Hippocampal Neurons
Source: PLoS One. 2015 Mar 20;10(3):e0120881. doi: 10.1371/journal.pone.0120881 (PMC4368532; doi:10.1371/journal.pone.0120881)
Supplement: S2 Text — (DOCX) [file pone.0120881.s012.docx]

**S2 Text. Morphological transformations produced by NMDA treatment: spines growth, collapse, and reappearance**

Imaging of live neurons during ischemia/reperfusion in slice cultures or in brain in vivo, demonstrated that dendrites undergo a characteristic morphological transformation, blebbing, (periodic dendritic swelling and shrinkage) within a few minutes of onset of an ischemia [[6](#_ENREF_6),[7](#_ENREF_7)]. These experiments also showed that spines could completely disappear during the ischemia and reappear only after a prolonged period of reperfusion, which depends on the severity of ischemia and the distance from the core ischemic region [[8](#_ENREF_8),[9](#_ENREF_9)]. In our experiments, we also frequently observed dendritic morphological transformations: swelling/shrinkage during or shortly after the NMDA treatment, which often partially recovered during the reperfusion. We were unable to establish an unbiased procedure for the quantification of the swelling process, partially because swelling often alternated with shrinkage (blebbing) and also because, without a cell volume marker, any quantification of morphological change would be inaccurate. Therefore, we describe the morphological changes produced by the NMDA treatment only qualitatively. S6 Fig. shows typical examples of these morphological changes produced by the NMDA treatment. Panels (A), (B), and (C) show full-frame images of dendritic segments from three experiments before, during, and after NMDA application. In most cases, we observed only moderate or no clear swelling/shrinkage during the application, but these became more pronounced later during the washout period (indicated by asterisks; see also Fig. 2, S2 Fig. and S3 Fig.). During the NMDA treatment we often observed an increase in spine fluorescence intensity and an apparent increase in spine size (S6 Fig., panels A-C, arrows). Later on, during the NMDA washout, in addition to a swelling/shrinkage of dendritic shafts, spines slowly became shorter and finally could completely disappear. This spine collapse contributed to the apparent decrease in spine fluorescent intensity observed in groups II–IV during the later periods of the washout after the NMDA treatment. S6 Fig., panels D, E show two additional examples of this dramatic alteration in spine morphology at higher magnification. During or after the NMDA treatment, there could also be a very short period of new filopodia generation (S6 Fig., panel D, arrow). Another interesting observation was that some collapsed spines could briefly reappear on the second NMDA treatment (S6 Fig., panel E, arrows), which we occasionally performed at the end of the washout period. Similar morphological changes were described in neurons during ischemia in vivo in different parts (core or penumbra) of the ischemic region [[7](#_ENREF_7),[8](#_ENREF_8),[9](#_ENREF_9)]. This similarity indicates that the NMDA procedure used in our study adequately represents an excitoxic/ischemic insult.

6. Murphy TH, Li P, Betts K, Liu R (2008) Two-photon imaging of stroke onset in vivo reveals that NMDA-receptor independent ischemic depolarization is the major cause of rapid reversible damage to dendrites and spines. J Neurosci 28: 1756-1772.

7. Zhang S, Boyd J, Delaney K, Murphy TH (2005) Rapid reversible changes in dendritic spine structure in vivo gated by the degree of ischemia. J Neurosci 25: 5333-5338.

8. Brown CE, Wong C, Murphy TH (2008) Rapid morphologic plasticity of peri-infarct dendritic spines after focal ischemic stroke. Stroke 39: 1286-1291.

9. Sigler A, Murphy TH (2010) In vivo 2-photon imaging of fine structure in the rodent brain: before, during, and after stroke. Stroke 41: S117-123.
