# Supplementary material for: Excitotoxic Insult Results in a Long-Lasting Activation of CaMKIIα and Mitochondrial Damage in Living Hippocampal Neurons
Source: PLoS One. 2015 Mar 20;10(3):e0120881. doi: 10.1371/journal.pone.0120881 (PMC4368532; doi:10.1371/journal.pone.0120881)
Supplement: S3 Text — (DOCX) [file pone.0120881.s013.docx]

**S3 Text. Statistical data for summary results shown in Fig. 3**

In group I, application of NMDA resulted in a persistent increase in fluorescence lifetime of Camui in both spines and dendritic shafts (0.064 ± 0.008 ns in spines and 0.077 ± 0.012 ns and in dendrites, p < 0.001 for both) and remained elevated for at least 55 min (0.05 ± 0.01 ns in spines and 0.048 ± 0.014 ns in dendrites, p < 0.05 for both) after the washout. Fluorescence intensity of Camui in spines persistently increased for ~40% (p < 0.05), while the fluorescence in dendritic shafts concomitantly decreased ~30% below the baseline (p < 0.05), resulting in a dramatic increase in the spine/dendrite fluorescence ratio (~100%). In group II, NMDA treatment produced only a transient increase in fluorescence lifetime of Camui (0.091 ± 0.015 ns in spines and 0.101 ± 0.009 ns in dendrites, p < 0.05 for both) immediately after the NMDA application but decreased to below the baseline (-0.034 ± 0.015 ns in spines and -0.025 ± 0.011 ns in dendrites at 30 min of the washout). Fluorescence intensity of Camui in spines remained elevated (~50%) for at least 30 min of NMDA washout but then gradually returned to the baseline by 45 min. Thus, group II neurons show an increase in Camui content in spines that persists longer than Camui activation. The Camui fluorescence in dendrite did not change significantly, and the spine/dendrite fluorescence ratio followed the general time course of the change in the fluorescence in spines.
