# Supplementary material for: Excitotoxic Insult Results in a Long-Lasting Activation of CaMKIIα and Mitochondrial Damage in Living Hippocampal Neurons
Source: PLoS One. 2015 Mar 20;10(3):e0120881. doi: 10.1371/journal.pone.0120881 (PMC4368532; doi:10.1371/journal.pone.0120881)
Supplement: S4 Text — (DOCX) [file pone.0120881.s014.docx]

**S4 Text. Camui mutants I205K and K42R did not prevent cluster formation**

We cannot rule out the possibility that the second tagged fluorophore in Camui (dVenus in addition to GFP) alters the regulation of CaMKII self-association; in both previous studies, only one tagged fluorophore (GFP) was used [[10](#_ENREF_10),[11](#_ENREF_11)]. Also, we cannot exclude that the variant of the catalytically dead mutant that we used ( K42R) might have a different effect in living cells [[12](#_ENREF_12)] in comparison to K42M CaMKII mutant used by [[11](#_ENREF_11),[13](#_ENREF_13)].

10. Hudmon A, Lebel E, Roy H, Sik A, Schulman H, et al. (2005) A mechanism for Ca2+/calmodulin-dependent protein kinase II clustering at synaptic and nonsynaptic sites based on self-association. J Neurosci 25: 6971-6983.

11. O'Leary H, Liu WH, Rorabaugh JM, Coultrap SJ, Bayer KU (2011) Nucleotides and phosphorylation bi-directionally modulate Ca2+/calmodulin-dependent protein kinase II (CaMKII) binding to the N-methyl-D-aspartate (NMDA) receptor subunit GluN2B. J Biol Chem 286: 31272-31281.

12. Lemieux M, Labrecque S, Tardif C, Labrie-Dion E, Lebel E, et al. (2012) Translocation of CaMKII to dendritic microtubules supports the plasticity of local synapses. The Journal of cell biology 198: 1055-1073.

13. Vest RS, O'Leary H, Bayer KU (2009) Differential regulation by ATP versus ADP further links CaMKII aggregation to ischemic conditions. FEBS Lett 583: 3577-3581.
