# Supplementary material for: Excitotoxic Insult Results in a Long-Lasting Activation of CaMKIIα and Mitochondrial Damage in Living Hippocampal Neurons
Source: PLoS One. 2015 Mar 20;10(3):e0120881. doi: 10.1371/journal.pone.0120881 (PMC4368532; doi:10.1371/journal.pone.0120881)
Supplement: S5 Text — (DOCX) [file pone.0120881.s015.docx]

**S5 Text. Clustering was not affected by mutations that prevented CaMKII autonomous state by either autophosphorylation of T286 or oxidation of CM280/281**

In experiments with WT, Camui clusters were formed in 90% of cells immediately after NMDA application and lasted to the end of experiments in 80% of these cells; In experiments with oxidation-deficient Camui (CM280/281VV), clusters formed in 89% of cells after NMDA, and they all retained clusters to the end of experiments; in experiments with autophosphorylation and oxidation-deficient Camui (T286A/CM280/281VV), clusters were formed in 92% of cells and persisted in all of these cells.
